# Targeting tumor-stroma communication by blocking endothelin-1 receptors sensitizes high-grade serous ovarian cancer to PARP inhibition

**DOI:** 10.1038/s41419-022-05538-6

**Published:** 2023-01-05

**Authors:** Piera Tocci, Celia Roman, Rosanna Sestito, Valeriana Di Castro, Andrea Sacconi, Ivan Molineris, Francesca Paolini, Mariantonia Carosi, Giovanni Tonon, Giovanni Blandino, Anna Bagnato

**Affiliations:** 1 Preclinical Models and New Therapeutic Agents Unit, Istituto di Ricovero e Cura a Carattere Scientifico (IRCCS), Regina Elena National Cancer Institute, Rome, Italy; 2 Translational Oncology Research Unit, IRCCS, Regina Elena National Cancer Institute, Rome, Italy; 3 Department of Life Science and System Biology, University of Turin, Turin, Italy; 4 Tumor Immunology and Immunotherapy Unit, IRCCS, Regina Elena National Cancer Institute, Rome, Italy; 5 Pathology Unit, IRCCS, Regina Elena National Cancer Institute, Rome, Italy; 6 Center for Omics Sciences (COSR) and Functional Genomics of Cancer Unit, Division of Experimental Oncology, IRCCS San Raffaele Scientific Institute, Milan, Italy; 7 Università Vita-Salute San Raffaele, 20132 Milan, Italy

**Keywords:** Cancer microenvironment, Ovarian cancer

## Abstract

PARP inhibitors (PARPi) have changed the treatment paradigm of high-grade serous ovarian cancer (HG-SOC). However, the impact of this class of inhibitors in HG-SOC patients with a high rate of TP53 mutations is limited, highlighting the need to develop combinatorial therapeutic strategies to improve responses to PARPi. Here, we unveil how the endothelin-1/ET-1 receptor (ET-1/ET-1R) axis, which is overexpressed in human HG-SOC and associated with poor prognosis, instructs HG-SOC/tumor microenvironment (TME) communication via key pro-malignant factors and restricts the DNA damage response induced by the PARPi olaparib. Mechanistically, the ET-1 axis promotes the p53/YAP/hypoxia inducible factor-1α (HIF-1α) transcription hub connecting HG-SOC cells, endothelial cells and activated fibroblasts, hence fueling persistent DNA damage signal escape. The ET-1R antagonist macitentan, which dismantles the ET-1R-mediated p53/YAP/HIF-1α network, interferes with HG-SOC/stroma interactions that blunt PARPi efficacy. Pharmacological ET-1R inhibition by macitentan in orthotopic HG-SOC patient-derived xenografts synergizes with olaparib to suppress metastatic progression, enhancing PARPi survival benefit. These findings reveal ET-1R as a mechanistic determinant in the regulation of HG-SOC/TME crosstalk and DNA damage response, indicating the use of macitentan in combinatorial treatments with PARPi as a promising and emerging therapy.

## Introduction

High-grade serous ovarian carcinoma (HG-SOC), in which over 90% of cases harbor TP53 mutations, is often diagnosed at advanced stages and is resistant to current therapies [1]. Among the anti-HG-SOC drug treatments, poly ADP-ribose polymerase inhibitors (PARPi) have shown promising clinical activities in the front-line management and in the maintenance treatment [2]. Despite the beneficial effects generated by their introduction into clinical treatments, eventual resistance to PARPi develops through mechanisms that have yet to be fully elucidated. Our understanding of the mechanisms underlying the therapeutic efficacy of PARPi is still evolving, including homologous recombination (HR)-dependent or HR-independent mechanisms that are largely dictated by the interactions between tumor cells and the tumor microenvironment (TME) [3–6]. Therefore, understanding the mechanisms of PARPi resistance in HG-SOC will enable the development of combination strategies to improve PARPi therapeutic efficacy.

Stromal cells and cancer cells exchange a multitude of autocrine/paracrine signals, including growth factors, cytokines and chemokines, which greatly affect both tumor and microenvironmental cell behavior [7, 8]. In such a complex tumor ecosystem, altered actionable drivers that engage molecular determinants involved in different oncogenic signaling routes may shape tumor/TME intercommunication. The molecular mechanism involved in HG-SOC/TME interactions and how this cross-talk impacts the PARPi response is currently not well characterized.

To date, different HG-SOC intrinsic mechanisms have been described for endothelin-1 (ET-1) that act on its receptors (ET-1R), the ET_A_ receptor (ET_A_R) and the ET_B_ receptor (ET_B_R), members of the G-protein coupled receptor (GPCR) family. Tumor-promoting events are regulated via ET_A_R, while TME-associated functions are mainly regulated via ET_B_R [9]. In OC, the ET-1/ET-1R axis has been implicated in the interplay with a network of pathways that confers features associated with the acquisition of aggressive traits [9]. In this scenario, recent findings shed light on the ability of ET-1/ET-1R signaling to integrate pathways that allow cells to remodel the extracellular matrix (ECM) through protease-secreting invadopodia, fueling metastatic progression [10].

In the array of oncogenic mediators that are activated by ET-1R, the cotranscriptional factor Yes-associated protein (YAP) [11–14] has been associated with HG-SOC progression and poor prognosis [15–17]. YAP has been studied not only for its implication in the metastatic process [18, 19] and response to different anticancer therapies [12, 13, 20–22], but also for its emerging activity in stromal elements, rewiring their phenotype and behavior, to generate a more corrupted TME [23–27]. Interestingly, mutant p53 (mutp53) activity is involved in the alliance between the ET-1/ET_A_R axis and YAP signaling in HG-SOC [12]. In these cells, the scaffold protein β-arrestin1 (β-arr1) acts as a nuclear linker that builds up a highly specific ET-1-driven transcriptional program [28–30], activating mutp53/YAP-dependent gene transcription [12].

An expanding body of evidence proves that YAP and mutp53, in addition to cooperating with each other [31–33], may interact with other transcription factors and cofactors at the transcriptional level, including those activated by hypoxia [34–37]. Functionally, HG-SOC cells exposed to hypoxia undergo profound rewiring, allowing them to metastasize through the activity of hypoxia-inducible factor (HIF-1α), the major hypoxia sensing transcription factor [3, 4]. In addition to hypoxic conditions, different signaling pathways, including ET-1, can induce HIF-1α activity [38, 39]. Interestingly, in the entourage of either YAP or mutp53 transcriptional allies, HIF-1α has been identified [34–37]. However, both the regulation and the functional alliance of YAP and HIF-1α in mutp53 ovarian cancer remain poorly defined. Therefore, a deeper understanding of the mechanisms acting both in the tumoral and the TME contexts, as instructed by ET-1 signaling, which implicates the integration of mutp53, YAP and HIF-1α activities, may open new therapeutic strategies to sensitize to PARPi.

In this study, we aimed to elucidate the impact of the ET-1R-driven mutp53/YAP/HIF-1α network, as a heterologous communication hub, on HG-SOC and stromal cell behavior. Based on the RNA-seq and TCGA data, as well as the analysis of patient-derived HG-SOC primary cultures, 3D cocultures, and orthotopic patient-derived xenografts (PDX), we report that repurposing macitentan, a dual ET_A_R/ET_B_R antagonist that is FDA-approved for treating pulmonary arterial hypertension [9], interferes with mutp53/YAP/HIF-1α hub-mediated tumor/stroma communication, and when combined with PARPi, elicits significant therapeutic efficacy in HG-SOC preclinical models. Thus, our findings reveal an approach that improves the response of HG-SOC to PARPi therapy.

## Materials and Methods

### Cells and chemical compounds

HG-SOC primary cells (PMOV10), carrying a germline missense mutation variant (R337T) on exon 9, were established and characterized as previously described [12]. The protocols for ascitic sample collection along with clinical information were approved by the Regina Elena institutional review board (IRB) and HG-SOC patients provided written informed consent. PMOV10 cells, the HG-SOC cell line OVCAR-3 (American Type Culture Collection (ATCC); VA, USA: HTB-161, *TP53* mutant R248Q), Human Umbilical Vein Endothelial Cells (HUVEC; Lonza, Switzerland) and the breast cancer cell line MDA-MB-468 (ATCC, VA, USA; HTB-132, *TP53* mutant R273H) were maintained as previously reported [12, 30]. Normal human lung fibroblasts (WI-38, CCL-75 ATCC) were cultured with Eagle’s minimum essential medium (EMEM) (30-2003 ATCC), supplemented with 10% FBS and 1% penicillin‒streptomycin. For experiments under hypoxic conditions, the cells were cultured as previously described [30]. Cell lines were routinely tested for mycoplasma contamination and authenticated by STR profiling. ET-1 (Sigma‒Aldrich, MO, USA; 100 nM), Macitentan (Actelion Pharmaceuticals, Ltd., Switzerland), also called ACT-064992 or *N*-[5-(4-Bromophenyl)-6-[2-[(5-bromo-2-pyrimidinyl)oxy]ethoxy]-4-pyrimidinyl]-*N*′-propyl-sulfamide, was added either 30 min before ET-1 or for 24 or 48 h at a dose of 1 μM. Olaparib (AZD2281, Selleckchem, United Kingdom), also called 4-[(3-[(4-cyclopropylcarbonyl)piperazin-1-yl]carbonyl)-4-fluorophenyl]methyl(2H)phthalazin-1-one, was added for 24 h or 48 h at 1 µM. For cell viability experiments, the concentration range was 0.5–30 µM. Cycloheximide was used at a dose of 100 μM and was purchased from Sigma‒Aldrich. Zibotentan (ZD4054, Selleckchem), also called N-(3-methoxy-5-methylpyrazin-2-yl)-2-(4-[1,3,4-oxadiazol-2-yl]phenyl) pyridine-3-sulfonamide, BQ123 (cyclo (-D-Trp–D-Asp–Pro–D-Val–Leu: Bachem, Switzerland) and BQ788 (N-cis-2,6-dimethylpiperidinocarbonyl-4-methyl-Leu-D-Trp (1-methoxycarbonyl)-D-Nle-OH Peninsula Laboratories LLC, San carlos, CA, United States), were added for 48 h at a dose of 1 µM.

### Immunoblotting (IB) and immunoprecipitation (IP)

Whole-cell lysates as well as nuclear and cytoplasmic fractions were obtained as previously reported [12]. Protein samples were electrophoresed on SDS-PAGE gels, as previously described [12]. Co-IP experiments for endogenous YAP and HIF-1α were carried-out as previously reported [12]. Bands were visualized using the enhanced chemiluminescence (ECL) detection (Bio-Rad, CA, USA). Information regarding antibodies (Abs) used in this research is detailed in Supplementary Table 1 included in the Supplementary Information.

### Immunofluorescence (IF) assay

Cells were fixed, permeabilized and blocked as previously published [12] and incubated overnight at 4 °C with primary Abs. Then, cells were incubated for 2 h in the dark with the secondary Abs. Nuclei were counterstained with DAPI (Bio-Rad) for 15 min. Slides were visualized at 64X magnification as previously described [12]. A minimum of 200 cells per condition was analyzed using the ImageJ program.

### Proximity ligation assay (PLA)

Cells were fixed, permeabilized and blocked as previously published [13]. After, the cells were incubated overnight at 4 °C with primary Abs YAP (G-6) (1:10, cat. no. 376830, Santa Cruz, TX, USA) and HIF-1α (1:10, cat. C166867, LS BIO, WA, USA). The PLA was performed as indicated by the manufacturer. Fluorescence signals were captured with a Leica DMIRE2 microscope and quantified with ImageJ program. At least 50 nuclei per condition were analyzed.

### Chromatin immunoprecipitation (ChIP) assay

Chromatin was analyzed from 5 × 10^6^ cells by ChIP, as previously reported [12]. Co-immunoprecipitated DNA was analyzed by PCR. The primers used for ChIP experiments are listed in Supplementary Table 2 included in the Supplementary Information.

### ELISA

PMOV10 cells, HUVECs and WI-38 fibroblasts silenced or not for YAP or HIF-1α were serum-starved for 24 h and then stimulated with ET-1 or hypoxia or treated with macitentan and olaparib, either alone or in combination for 48 h. The release of ET-1 and VEGF was measured in the cell conditioned media (CM) by Quantikine ELISA kits for human Endothelin or human VEGF (R&D Systems, MN, USA) following manufacturer’s instructions.

### Three-dimensional HG-SOC spheroid and HG-SOC/EC spheroid sprouting assay

A three-dimensional spheroid sprouting assay was performed with PMOV10 cell spheroids and PMOV10/HUVEC hybrid spheroids by plating 100 cells per well for 48 h in 3D Culture Qualified 96-well plates. Then, spheroids were embedded into the matrix (Cultrex; Trevigen, MD, USA) and properly stimulated. Spheroids were photographed before and after treatment using a ZOE Fluorescent Cell Imager (Bio-Rad). Quantification of sprout length was performed using the ImageJ program.

### Transendothelial migration assay

HUVEC were seeded (5 × 10^4^ cells) on 8.0-μm pore membranes of a BD Invasion Chamber (BD Biosciences, NJ, USA) and left to form a monolayer for 24 h at 37 °C. PMOV10 cells silenced or not for YAP, HIF-1α or p53 underwent red staining (Sigma‒Aldrich) for 15 min at 37 °C, then were plated onto the HUVEC, and were subsequently stimulated with ET1 and/or macitentan, olaparib and/or macitentan, and allowed to invade for 24 h. Transmigrated cells were analyzed at 20X magnification with a ZOE Fluorescent Cell Imager.

### Migration assay with co-cultures of HG-SOC/EC and HG-SOC/activated fibroblasts

HUVEC and WI-38 fibroblasts (4 × 10^4^), stained in red and PMOV10 cells (7 ×10^4^), stained in green (Sigma‒Aldrich), silenced or not for YAP, HIF-1α and p53, were cultured in a well separated by an insert (Culture–Insert 2 Well, Ibidi GmbH, Germany) until they reached confluence, when the insert was removed and pictures were taken at time 0 (T0). Then cells were stimulated with ET-1, PMOV10 CM, HUVEC CM or fibroblast CM, or were treated with macitentan for 24 h when pictures were taken (T24 h) with a ZOE Fluorescent Cell Imager. Cell-free gap area was measured with the ImageJ program.

### TCGA data analysis

Computational data from The Cancer Genome Atlas (TCGA) of HG-SOC patients was obtained from Agilent gene expression level 3 TCGA data from GDAC Firehose [40]. Data are lowess normalized (cy5/cy3), log2 transformed and collapsed by gene symbol. Survival analyses were generated through the Kaplan‒Meier method, and a log-rank test was employed to establish the distance between the curves. The combined expression was obtained considering the average values of the genes. High and low expression were then evaluated considering positive and negative z-scores, respectively. A Cox hazard regression model was also included with hazard risk (HR), confidence interval at 95% and a p-value as an estimate of the significance of this interval. A *p*-value of <0.05 was considered significant. MATLAB (The Math Works) was used for computational analysis. GSEA: Public available data used in this article were obtained from GSEA Gene Set Enrichment Analysis (https://www.gsea-msigdb.org/gsea/index.jsp).

### RNA sequencing of patient-derived HG-SOC cells

Total RNA was isolated from PMOV10 cells using the RNeasy Quick Start kit (Qiagen, Germany) according to manufacturer’s instructions. Before starting with library prep, RNA quality was determined by running samples on the 4100 TapeStation system (Agilent, CA, USA). Only RNA samples with RIN above 7 were used in the next steps. To generate the NGS libraries, we used the TruSeq Stranded mRNA kit (Illumina, CA, USA), starting with 100 ng of total RNA. GSEA preranked analysis of signaling pathways from Hallmark and Kyoto Encyclopedia of Genes and Genomes (KEGG) gene sets was performed using the Java version of the software (gsea2–2.2.3.jar; software.broadinstitute.org/gsea/), with log2FC obtained from RNA-Seq contrasts and standard parameters. RNA-Seq data have been uploaded to the Gene Expression Omnibus (GEO) database (https://www.ncbi.nlm.nih.gov/geo/), with accession number GSE196065.

### Cytokine and chemokine assay

PMOV10 cells were seeded (15 × 10^4^) and subsequently silenced or not for YAP, HIF-1α or p53 for 72 h and either stimulated or not with ET-1 for 24 h. Supernatants were collected after 24 h and centrifuged for 10 min at 1000 g to eliminate cell debris and thereafter diluted for the Luminex assay. Cytokines and chemokines were analyzed using a Bio-Plex Pro Human Cytokine Screening 48-Plex panel (Bio-Rad) according to the manufacturer’s instructions. The cytokine/chemokine contents of each well were identified and quantified against standard samples using a Bio-Plex Magpix apparatus (Bio-Rad).

### Animals

Six- to eight-week-old female athymic nude-CD1 nu+ /nu+ mice (Envigo Laboratories, IN, USA) were housed under pathogen-free conditions. Procedures involving animals and their care were conducted with the permission from the IRCCS Regina Elena Cancer Institute Animal Care and Use Committee and the Italian Ministry of Health (D.lgs 26/2014, authorization number 1083/2020PR, issued 5 November 2020 by Ministero della Salute).

### PDX studies

HG-SOC-PDX were established by intraperitoneal (i.p.) injection of a tumor suspension of freshly isolated PMOV10 cells (2.5 × 10^6^ in 200 μl PBS) in the peritoneal cavity of nude mice, as previously reported [12]. After 7 days of latency, mice were randomly subdivided into four groups (*n* = 8), undergoing the following treatments: control (CTR; vehicle) versus macitentan (MAC; 30 mg/kg/oral daily) and/or olaparib (50 mg/kg/oral daily), in monotherapy or in combination therapy. The control group underwent the same schedule as those mice given the active drug. Mice were monitored daily and subsequently euthanized when they presented signs of distress due to disease progression (visible abdominal swelling, hemorrhagic ascites, and palpable abdominal tumor masses. To follow-up the combination therapy biosafety, we monitored the tolerability of the drugs. Notably, we did not observe body weight loss in all treatment groups. Following 5 weeks, mice were euthanized by cervical dislocation and intraperitoneal tumor nodules were taken throughout the peritoneal cavity. Values represent the mean of the number of visible metastases ± SD of 8 mice in each group from two independent experiments. The drug combination effect was analyzed by calculating the coefficient of drug interaction (CDI) based on the Chou-Talalay method [41]. CDI < 1 indicates that the drugs are synergistic, CDI = 1 that are additive.

### Statistical analysis

With the exception of the mice model studies, data points represent the mean of three independent experiments performed in triplicates with standard deviation (SD). Student’s t-test was utilized to compare two groups of independent samples and to estimate the sample size of mice chosen for each treatment group. Statistical analysis of cell survival within time for each concentration of treatment was performed using the two-way ANOVA test. All data analyses were conducted in GraphPad Prism v8.0 software. For the present study, we used cell cultures with a normal distribution and similar variance between groups.

More information on materials and methods is available in the section Supplementary materials and methods of Supplementary information.

## Results

### ET-1 drives HIF-1α and YAP nuclear accumulation, and the *YAP1/EDNRA/HIF1A* signature is correlated with poor prognosis in HG-SOC patients

**Fig. 1:**
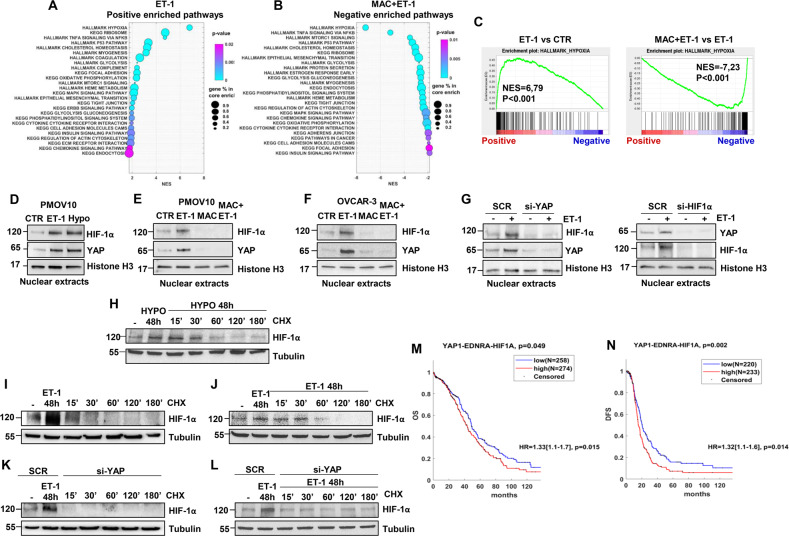
ET-1 drives HIF-1α and YAP nuclear accumulation, and the *YAP1/EDNRA/HIF1A* signature is correlated with poor prognosis in HG-SOC patients. **A**, **B** Bubble charts of the top 26 hallmark and KEGG gene sets enriched in HG-SOC patient-derived (PD) primary cultures of HG-SOC cells (PMOV10) upon ET-1 (100 nM) stimulation for 24 h in comparison to untreated cells (CTR) (**A**) or downregulated upon macitentan (MAC, 1 µM) plus ET-1 treatment for 24 h in comparison to ET-1-treated cells (**B**). **C** ET-1 enriches the hypoxia gene signature in PMOV10 cells. GSEA in cells stimulated with ET-1 or treated with MAC plus ET-1 with the normalized enrichment score (NES). **D–F** Immunoblotting (IB) analysis for HIF-1α and YAP in nuclear extracts of PMOV10 (**D**, **E**) and OVCAR-3 (**F**) cells stimulated with ET-1 for 2 h (**D–F**) or treated with MAC (**E**, **F**). Histone H3 represents the loading control. **G** IB analyses of HIF-1α and YAP protein expression in nuclear extracts of PMOV10 cells stimulated with ET-1 for 2 h and transfected for 72 h with a siRNA control (SCR) or siRNA specific for YAP (si-YAP; *left*) or HIF-1α (si-HIF-1α, *right*). **H**-**J** PMOV10 cell extracts stimulated with ET-1 (**I**, **J**) or hypoxia (**H**) for 48 h were treated with cycloheximide (CHX: 100 µM) for the indicated times. Tubulin represents the loading control. **K**, **L** PMOV10 cell extracts silenced for YAP and stimulated (**L**) or not stimulated (**K**) with ET-1 for 48 h were treated with CHX for the indicated times. Tubulin represents the loading control. Representative images of blots of 3 independent experiments are shown in D-L. **M**, **N** Overall survival (OS) and disease-free survival (DFS) analysis of HG-SOC patients with high and low combined gene expression levels of *YAP1*, *EDNRA* and *HIF1A* (OS (**M**): *p* = 0.049; DFS (**N)***: p* = 0.002). The combined expression was obtained considering the average values of the genes. High and low expression were then evaluated in consideration of positive and negative z scores, respectively. Differences between curves were assessed by the log-rank test. A Cox hazard regression model was also included with hazard risk (HR), confidence interval at 95% and a *p* value as an estimate of the significance of this interval. A *p* value less than 0.05 (<0.05) was considered significant (OS HR: *p* = 0.015; DFS HR: *p* = 0.014).

To explore the contribution of ET-1/ET-1R axis activation toward the transcriptional traits of HG-SOC cells, we monitored the global transcriptome in a preclinical relevant model of patient-derived (PD) primary cultures of HG-SOC cells (PMOV10) harboring *TP53* mutations [12]. We stimulated PMOV10 cells with ET-1, treated them with the ET-1R antagonist macitentan, and then analyzed the transcriptome with RNA-Seq. Gene set enrichment analysis (GSEA) revealed a list of signaling pathways enriched upon ET-1 stimulation and downregulated following macitentan treatment (Fig. 1A, B). The most significant differentially expressed networks belonged to hypoxia-related pathways (Fig. 1A–C). We have previously shown that in response to ET-1 axis activation, YAP and mutp53 are concurrently engaged in a nuclear platform [12] that may be utilized by other transcription factors, including HIF-1α. Indeed, previous results have shown the ability of YAP to form a complex with HIF-1α [34, 42, 43], providing a strong rationale to investigate the molecular mechanisms underlying HIF-1α/YAP interdependence.

To verify whether HIF-1α may integrate ET-1-mediated YAP activity, we utilized PMOV10 and OVCAR-3 cells, both of which express ET_A_R and ET_B_R (Supplementary Fig. 1A). In these cells, ET-1, to an extent comparable to hypoxia, guided the ET_A_R-dependent nuclear compartmentalization of HIF-1α and concomitantly induced YAP nuclear accumulation (Fig. 1D–F and Supplementary Fig. 1B, C), whereas macitentan prevented both of these effects (Fig. 1E, F and Supplementary Fig. 1C). It has previously been reported that YAP activation may be induced by hypoxia in several solid tumors [35, 44], thus we analyzed whether ET-1 can coordinate a regulatory loop between HIF-1α and YAP in HG-SOC cells. Interestingly, silencing of either HIF-1α or YAP abolished the ET-1-induced accumulation of YAP and HIF-1α, respectively, highlighting the interdependency of ET-1-induced HIF-1α/YAP nuclear co-accumulation in HG-SOC cells (Fig. 1G and Supplementary Fig. 1D, E). In parallel with decreased YAP phosphorylation, ET-1 reduced LATS1 phosphorylation (Supplementary Fig. 1F). Moreover, LATS1 silencing blunted the impact of ET-1 on YAP/HIF-1α nuclear co-accumulation (Supplementary Fig. 1G, L), suggesting that the ET-1R pathway regulates YAP/HIF-1α nuclear localization, by interfering with LATS kinase activity.

Additionally, YAP depletion impaired ET-1-induced HIF-1α protein expression and the expression of VEGF, one of the major HIF-1α target genes (Fig. 1H and Supplementary Fig. 1H). The upregulation of HIF-1α is achieved by inhibiting proteasomal degradation through YAP, which is capable of binding to HIF-1α and sustaining HIF-1α protein stability [34]. *De novo* protein synthesis analysis in cells exposed to different cycloheximide kinetics before and after ET-1 or upon hypoxia stimulation (Fig. 1H–J) revealed that HIF-1α protein stability was enhanced in those cells stimulated with ET-1, with an effect that was comparable to hypoxia (Fig. 1H–J). In these cells, YAP silencing abrogated HIF-1α stability with and without ET-1 stimulation (Fig. 1K–L), indicating that YAP regulates ET-1-induced HIF-1α expression by enhancing the stability of HIF-1α.

Finally, to measure the clinical impact generated by the integration of the ET-1R axis, YAP and HIF-1α gene expression on the survival of HG-SOC patients, we evaluated the prognostic significance associated with their combined expression in The Cancer Genome Atlas (TCGA) data set [40]. The higher combined expression of *YAP1*, *EDNRA*, and *HIF1A* significantly correlated with a dismal prognosis of HG-SOC patients in terms of overall survival (OS) [hazard ratio (HR) = 1.33 (1.1–1.7), *P* = 0.015] (Fig. 1M) and disease-free survival (DFS) analysis [HR = 1.32 (1.1–1.6), *P* = 0.014] (Fig. 1N) when compared to those with a low-expression gene signature. Thus, the *YAP1*/*EDNRA*/*HIF1A* combined gene expression emerges as a predictive signature of poor prognosis in HG-SOC patients.

### ET-1 instructs the formation of a mutp53/YAP/HIF-1α nuclear complex regulating both proinvasive and angiogenic factor secretion

**Fig. 2:**
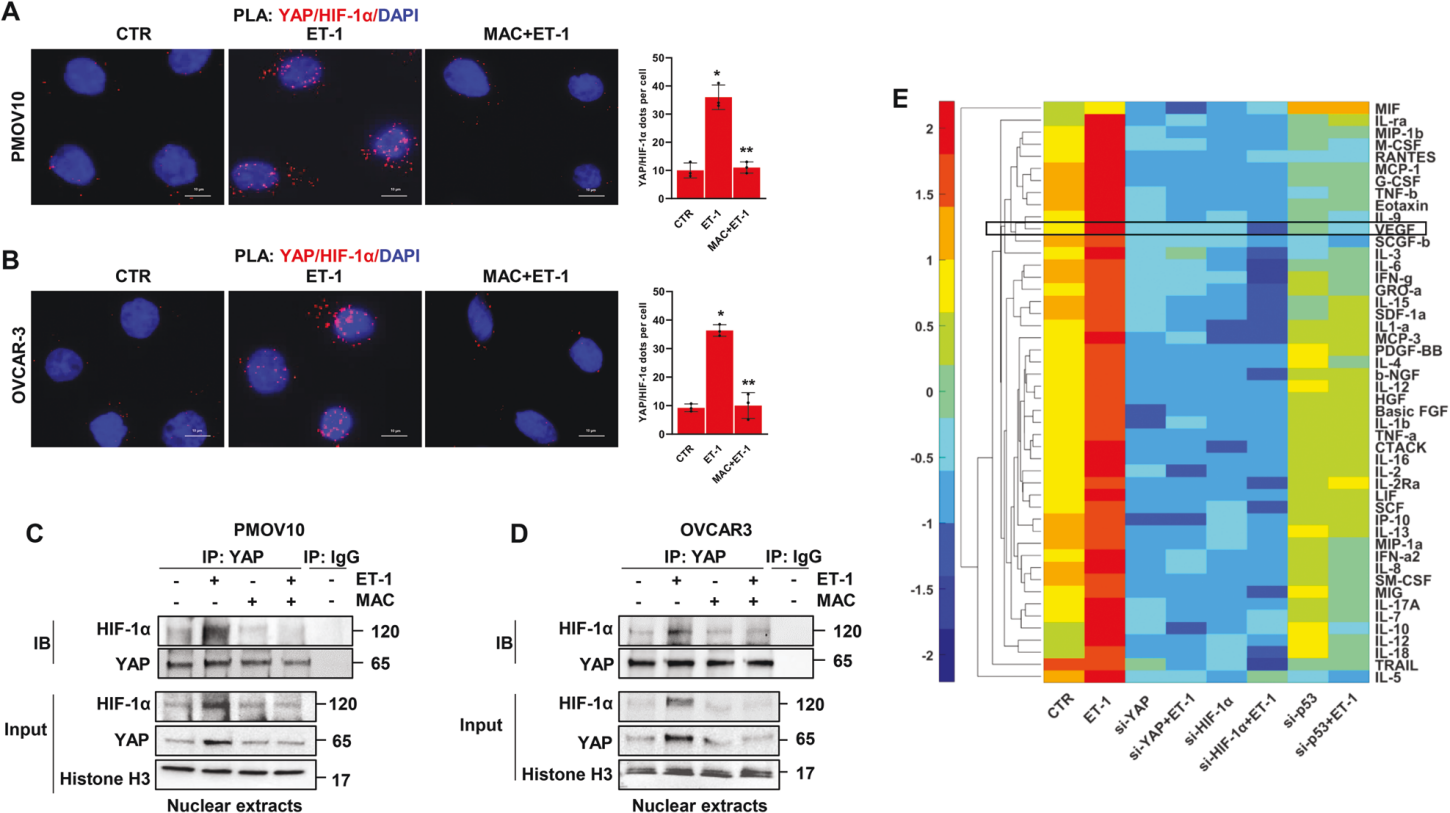
ET-1 triggers the formation of YAP and HIF-1α nuclear complex, which regulates proinvasive and angiogenic factor secretion. **A**, **B** Representative images of proximity ligation assay (PLA) detection of YAP and HIF-1α protein complexes (red signals) in ET-1-stimulated PMOV10 (**A**) and OVCAR-3 (**B**) cells and/or treated with MAC for 2 h. DAPI nuclear staining (blue). Scale bar, 10 µm. The right graphs show the PLA quantification. Bars are means ± SD (*p < 0.001 vs. CTR; **p < 0.0009 vs. ET-1; *n* = 3). **C**, **D** Nuclear extracts of PMOV10 (**C**) or OVCAR-3 (**D**) cells stimulated with ET-1 and/or MAC for 2 h were immunoprecipitated (IP) for YAP using anti-YAP or anti-IgG and IB using anti-YAP and anti-HIF-1α. Histone H3 represents the loading control. Representative images of blots of 3 independent experiments are shown. **E** Hierarchical clustering of the mean standardized fluorescence intensity of 48 cytokines in PMOV10 cell conditioned media (CM) stimulated or not with ET-1 for 24 h or silenced for YAP, HIF-1α or p53 for 72 h. Factors were clustered based on Euclidean distance.

Having observed the nuclear co-accumulation of HIF-1α and YAP in response to ET-1 stimulation and considering the interplay between YAP and mutp53 [12, 31–33] and p53 and HIF-1α [45], we envision a scenario in which ET-1 signaling may guide the physical interaction and functional cooperation among these mediators. To begin validating this hypothesis, we employed in situ proximity ligation assay (PLA) technology and detected the presence of YAP/HIF-1α nuclear complexes in HG-SOC cells. While ET-1 increased the presence of this complex in PMOV10 (Fig. 2A) and OVCAR-3 cells (Fig. 2B), macitentan treatment strongly impaired its formation (Fig. 2A, B). Similar results were obtained by co-immunoprecipitation (co-IP) analysis in PMOV10 and OVCAR-3 cells (Fig. 2C, D). Considering that the duration and localization of receptor signaling may be fine-tuned by β-arr1 [46, 47], we observed that ET-1 stimulus enhanced its interaction, not only with HIF-1α and YAP, but also with β-arr1 and mutp53, thereby leading to the formation of a distinct nuclear network (Supplementary Fig. 2A). These results were validated by reciprocal co-IP analysis using HIF-1α immunoprecipitates (Supplementary Fig. 2B). Thus, all of the components of this complex were needed, such that the absence of one of the components led to the disassembly of the complex (Supplementary Figs. 1I–K and 2A). We then investigated whether or not the ET-1R-driven formation of this multiprotein complex can affect the release of cytokines, chemokines, and growth factors in the conditioned media (CM) collected from HG-SOC cells. Analysis of several factors, namely, MIF, IL-ra, MIP-1β, M-CSF, RANTES, MCP-1 (MCAF), G-CSF, TNF-β, eotaxin, IL-9, VEGF, SCGF-β, IL-3, IL-6, IFN-γ, GRO-α, IL-15, SDF-1α, IL-1α, MCP-3, PDGF-BB, IL-4, β-NGF, IL-12, HGF, basic FGF, IL-1β, TNF-α, CTACK, IL-16, IL-2, IL-2R α, LIF, SCF, IP10, IL-13, MIP-1 α, IFN- α 2, IL-8, SM-CSF, MIG, IL-17A, IL-7, IL-10, IL-12, IL-18, TRAIL, and IL-5, demonstrated an upregulation of the factor concentrations in the media of cells upon ET-1 stimulation, when compared to untreated cells. Conversely, YAP, HIF-1α or p53 depletion reduced the concentrations of these soluble factors. Notably, VEGF appeared to be one of the most released factors in response to ET-1 (Fig. 2E). Altogether, these findings indicate that the ET-1/ET-1R-driven mutp53/YAP/HIF-1α complex modulates a repertoire of proinvasive and angiogenic cues, including VEGF.

### The ET-1R-driven mutp53/YAP/HIF-1α network engages a cooperative transcriptional program driving cell invasion and transendothelial migration

**Fig. 3:**
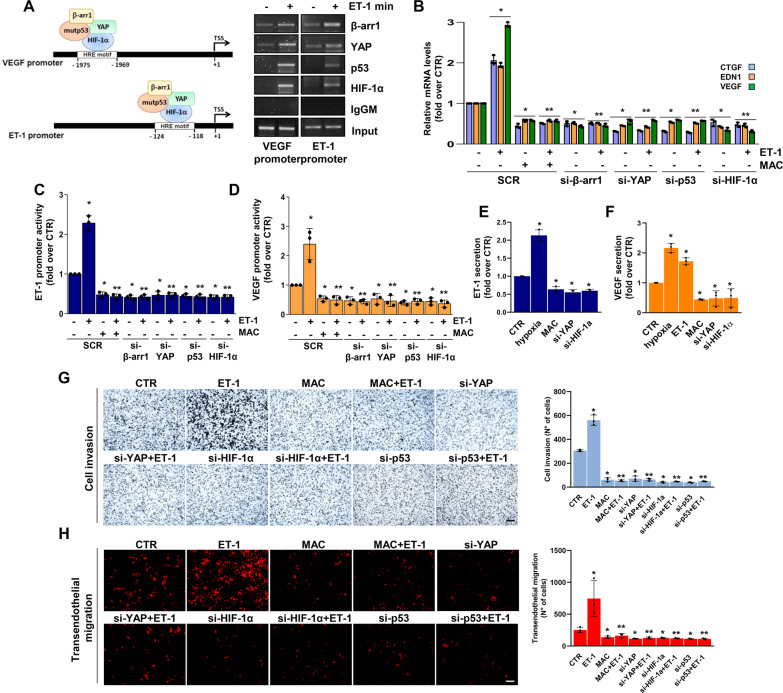
mutp53/YAP engages HIF-1α to mediate the ET-1-induced transcriptional cooperation that promotes HG-SOC cell invasion and transendothelial migration. **A** The recruitment of β-arr1, YAP and p53 on the HIF-1α binding motif (HRE motif) in the VEGF and ET-1 promoters of PMOV10 cells stimulated with ET-1 for 2 h was measured by chromatin immunoprecipitation (ChIP) followed by PCR. Anti-IgG mouse Ab (IgGM) was used as a control for all ChIP reactions. **B** qRT-PCR analysis of YAP/HIF-1α mRNA target genes in PMOV10 cells stimulated with ET-1 and treated with MAC for 24 h or silenced for β-arr1, YAP, p53 and HIF-1α for 72 h. Bars are means ± SD (*p < 0.0007 vs. CTR; **p < 0.0002 vs. ET-1; *n* = 3). **C**, **D** ET-1 (C) and VEGF (**D**) promoter activity in PMOV10 cells silenced as in B, cotransfected with ET-1 or VEGF promoter-luc constructs, and treated with ET-1 and/or MAC for 24 h. Bars are means ± SD (*p < 0.02 vs. CTR; **p < 0.004 vs. ET-1; *n* = 3). **E**, **F** ELISA for ET-1 (**E**) and VEGF **(F**) released by PMOV10 cells with or without YAP or HIF-1α silencing for 72 h and stimulated with ET-1 or hypoxia or treated with MAC for 24 h. Bars are means ± SD (*p < 0.05 vs. CTR; *n* = 3). **G** Invasion assay of PMOV10 cells stimulated or not with ET-1, treated with MAC for 24 h, or transfected with SCR, si-YAP, si-HIF-1α or si-p53 for 72 h. Representative images of invading cells were photographed (scale bar: 100 µm, magnification 20X) (*left panels*) or counted (*right graph*). Bars are means ± SD (*p < 0.0007 vs. CTR; **p < 0.0002 vs. ET-1; *n* = 3). **H** Transendothelial migration assay of PMOV10 cells silenced or not for YAP, HIF-1α or p53 for 72 h and stimulated with ET-1 and/or MAC for 24 h. Representative images of transmigrated cells were photographed (scale bar: 100 µm, magnification 20X) (*left panels*) or counted (*right graph*). Bars are means ± SD (*p < 0.05 vs. CTR; **p < 0.03 vs. ET-1; n = 3).

To determine the transcriptional functionality of the identified YAP/mutp53/β-arr1/HIF-1α nuclear complex, chromatin immunoprecipitation (ChIP) analysis was performed. The results illustrated that upon ET-1 stimulation, β-arr1/YAP/mutp53 were recruited to the hypoxia-responsive elements (HRE) of the *ET-1* and *VEGF* gene promoters [30], where HIF-1α was concomitantly anchored to activate a cooperative transcriptional program (Fig. 3A). Consequently, qRT-PCR revealed that ET-1 stimulation increased YAP/HIF-1α target gene expression, such as endothelin-1 (*EDN1*), *VEGF*, and connective tissue growth factor (*CTGF*) (Fig. 3B and Supplementary Fig. 3A–E), and this effect was prevented either by β-arr1, YAP, mutp53 and HIF-1α depletion, or upon macitentan treatment (Fig. 3B and Supplementary Figs. 1I–L, and 3A–E). Along with these results, ET-1 stimulation induced the upregulation of ET-1 and VEGF promoter activity, which was curbed upon the exclusion of each element of the transcriptional competent complex or by macitentan (Fig. 3C, D and Supplementary Fig. 3F, G). The activation of the ET-1 axis, similar to hypoxic stimulus, favored a greater release, not only of VEGF, but also of ET-1. The silencing of YAP or HIF-1α, as well as macitentan, strongly reduced the VEGF and ET-1 released by HG-SOC cells (Fig. 3E, F). Altogether, these results suggest that, downstream of ET-1R, the mutp53/YAP/HIF-1α transcriptional complex represents a multiprotein machine to deploy a cooperative program that impacts the release of different factors, including ET-1 itself, thus amplifying a self-feeding ET-1/ET-1R circuit.

To explore the involvement of the ET-1-driven mutp53/YAP/HIF-1α signaling circuit in the acquisition of aggressive features, we performed a transwell cell invasion assay and observed that macitentan, similar to YAP, HIF-1α or p53 depletion, hampered the ET-1-mediated invasive behavior of HG-SOC cells (Fig. 3G). Remarkably, HG-SOC metastatic dissemination requires transendothelial migration, a process by which tumor cells cross the endothelial cell (EC) barrier and extravasate from blood vessels. While ET-1 stimulation significantly promoted HG-SOC cell movement across the EC barrier, YAP, HIF-1α or p53 silencing, as well as macitentan treatment, significantly reduced these effects (Fig. 3H), thereby strengthening the major role of the ET-1-mediated signaling network in HG-SOC cell invasion and metastatic extravasation.

### The ET-1-mediated p53/YAP/HIF-1α circuitry fuels the signal reciprocity between HG-SOC cells and endothelial cells

**Fig. 4:**
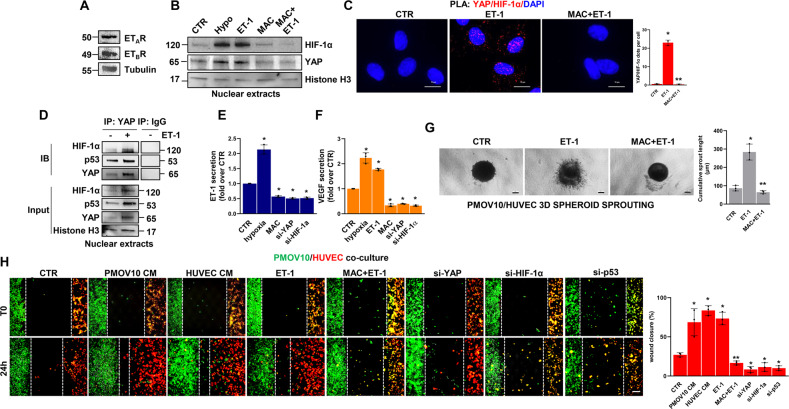
The ET-1-triggered p53/YAP/HIF-1α network enhances HG-SOC/endothelial cell cross-talk. **A** IB analysis of ET_A_R and ET_B_R protein expression in total HUVEC extracts. Tubulin represents the loading control. **B** IB analysis of YAP and HIF-1α protein expression in nuclear extracts of HUVEC stimulated with ET-1 and/or MAC or hypoxia for 2 h. Histone H3 represents the loading control. **C** Representative images of PLA detection of YAP and HIF-1α protein complexes (red signals) in HUVEC stimulated with ET-1 and/or MAC for 2 h. Nuclei are stained blue (DAPI). (Scale bar: 10 µm, magnification 64X). The right graph shows the PLA quantification. Bars are means ± SD (*p < 0.0002 vs. CTR; **p < 0.0002 vs. ET-1; *n* = 3). **D** Nuclear extracts of HUVEC stimulated with ET-1 for 2 h were IP for YAP, using anti-YAP, or anti-IgG and IB, using anti-YAP, anti-p53 and anti-HIF-1α. Histone H3 represents the loading control. Representative images of blots of 3 independent experiments are shown. **E**, **F** ELISA for ET-1 (**E**) and VEGF (**F**) released by HUVEC silenced or not for YAP or HIF-1α for 72 h and stimulated with ET-1 or hypoxia, or treated with MAC for 24 h. Bars are means ± SD (*p < 0.04 vs. CTR; *n* = 3). **G** Representative images of 3D hybrid PMOV10/EC spheroids sprouting into the surrounding matrix upon stimulation with exogenous ET-1 and/or MAC for 48 h (scale bar: 100 µm, magnification 10X)*. Right graph*, quantification of cumulative sprout length (µM). Bars are means ±SD (*p < 0.002 vs. CTR; **p < 0.002 vs. ET-1 treated cells; *n* = 3). **H** Cocultured cells with or without YAP, HIF-1α or p53 silencing were stained green (PMOV10) and red (HUVEC) and then treated with ET-1 and/or macitentan or with PMOV10 CM or HUVEC CM for 24 h. Representative images of cells were photographed both at T0 and upon treatment for 24 h (scale bar: 100 µm, magnification 20X). The right graph indicates the percentage of gap closure. Bars are means ± SD (*p < 0.02 vs. CTR; **p < 0.0004 vs. ET-1; *n* = 3).

In view of the role of ET-1 and YAP signaling in establishing an autocrine-paracrine signaling route between tumor cells and neighboring ECs [24, 38, 39], we examined whether ET-1 can guide the activation of the p53/YAP/HIF-1α signaling complex within EC. IB analysis performed in human umbilical vein endothelial cells (HUVEC) expressing both ET_A_R and ET_B_R (Fig. 4A), revealed that ET-1 stimulation induced YAP dephosphorylation in a time-dependent manner (Supplementary Fig. 4A, B) and favored YAP and HIF-1α nuclear accumulation (Fig. 4B). These effects were reversed by macitentan treatment (Fig. 4B and Supplementary Fig. 4B). Furthermore, PLA analysis documented the formation of YAP/HIF-1α nuclear complexes in ET-1-stimulated cells, which were disrupted upon treatment with macitentan (Fig. 4C). In addition, co-IP analysis revealed the ET-1-guided formation of a nuclear complex comprising not only YAP and HIF-1α, but also p53, whose functionality is involved in EC-related angiogenic effects [48, 49] (Fig. 4D).

The ET-1 axis, mimicking hypoxia, sustained the release of VEGF and ET-1, an effect prevented by macitentan and by YAP and HIF-1α silencing (Fig. 4E, F and Supplementary Fig. 4C, D). Moreover, ET-1-stimulated or EC CM-treated HG-SOC cells displayed a higher tumor migration rate, that was inhibited by macitentan or EC CM that had been previously depleted of YAP, HIF-1α or p53 (Supplementary Fig. 4F). Next, to examine whether ET-1-mediated soluble factor secretion by HG-SOC cells can boost the angiogenic behavior of EC, we performed migration and tube formation assays, showing that the stimulation of EC with HG-SOC CM, similar to the effect with exogenous ET-1, impacted their ability to migrate and form vascular structures. The treatment of EC with CM from HG-SOC cells, previously depleted for YAP expression, HIF-1α, p53 or pretreated with macitentan, prevented these effects (Supplementary Fig. 4G, H). The capability of ET-1 signaling to instruct reciprocal intercellular communication between HG-SOC cells and EC was further analyzed in 3D hybrid spheroids, including PD HG-SOC cells and EC, in a matrix mimicking the ECM. In ET-1-stimulated spheroids, the length of sprouts invading the surrounding matrix was significantly enhanced, whereas spheroids treated with macitentan showed a reduced ability to invade (Fig. 4G). In addition, in HG-SOC/EC co-cultures, ET-1-stimulated cells tried to reach each other, establishing a gap closure of approximately 73%, similar to the effect generated by treating cells with HG-SOC and EC CM (gap closure of 69 and 84%, respectively; Fig. 4H). Interestingly, co-cultured HG-SOC/EC treated with macitentan or silenced for YAP, HIF-1α and p53 were almost unable to move. Altogether, these results suggest that the ET-1-mediated activation of the p53/YAP/HIF-1α circuit operating in both HG-SOC and EC contributes, through the bidirectional release of autocrine/paracrine factors, to the connection of tumor cells with stromal components that can be interrupted by macitentan.

### The ET-1-induced p53/YAP/HIF-1α communication hub promotes the connection between HG-SOC cells and activated fibroblasts

**Fig. 5:**
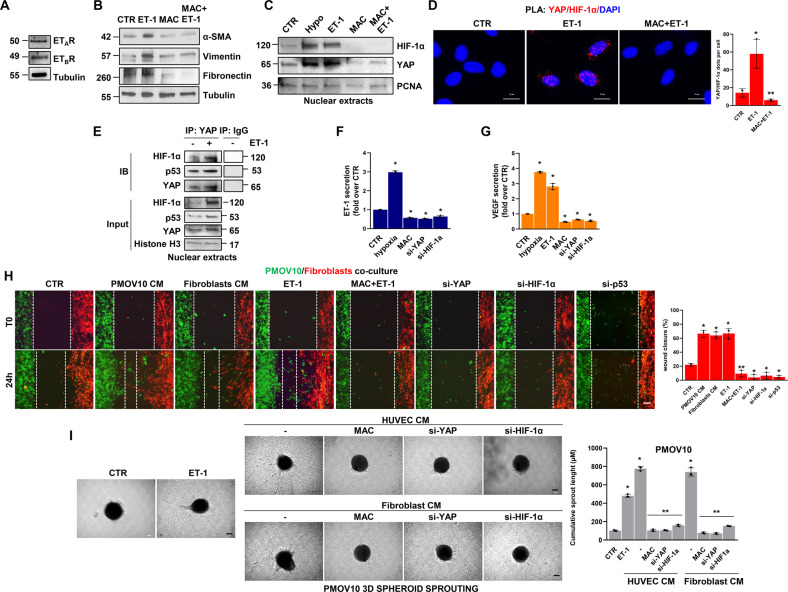
The ET-1-triggered p53/YAP/HIF-1α circuit mediates HG-SOC/activated fibroblast interplay. **A** IB analysis for ET_A_R and ET_B_R protein expression in total extracts of human fibroblasts. Tubulin is used as a loading control. **B** IB analysis for α-SMA, vimentin and fibronectin in total extracts of fibroblasts stimulated with ET-1 and/or MAC for 24 h. Tubulin is used as a loading control. **C** IB analysis of YAP and HIF-1α protein expression in nuclear extracts of fibroblasts stimulated with ET-1 and/or MAC or hypoxia for 2 h. PCNA was used as a loading control. **D** Representative images of PLA detection of protein complexes containing YAP and HIF-1α (red signals) in ET-1-stimulated fibroblasts and/or treated with MAC for 2 h. Nuclei are stained in blue (DAPI). (scale bar: 10 µm, magnification 64X). The right graph shows the PLA quantification. Bars are means ± SD (*p < 0.02 vs. CTR; **p < 0.006 vs. ET-1; *n* = 3). **E** Nuclear extracts of fibroblasts stimulated with ET-1 for 2 h were IP for YAP using anti-YAP or anti-IgG and IB using anti-YAP, anti-p53 and anti-HIF-1α. Histone H3 represents the loading control. Representative images of blots of 3 independent experiments are shown in A-C, and E. **F**, **G** ELISA for ET-1 (**F**) and VEGF (**G)** released by fibroblasts silenced or not silenced for YAP or HIF-1α and stimulated with ET-1 or hypoxia or treated with MAC for 24 h. Bars are means ± SD (*p < 0.0006 vs. CTR; *n* = 3). **H** Cocultured cells with or without YAP, HIF-1α or p53 silencing for 72 h were stained green (PMOV10) and red (fibroblasts) and then treated with ET-1 and/or macitentan or with PMOV10 CM or fibroblast CM for 24 h. Representative images of cells were photographed at T0 and upon treatment for 24 h (scale bar: 100 µm, magnification 20X). The right graph indicates the percentage of gap closure. Bars are means ± SD (*p < 0.0007 vs. CTR; **p < 0.0004 vs. ET-1; *n* = 3). **I** Representative images of 3D PMOV10 spheroids sprouting into the surrounding matrix upon stimulation with exogenous ET-1, HUVEC CM or fibroblast CM for 48 h, or upon stimulation with CM from fibroblasts or HUVEC treated with macitentan or silenced for YAP or HIF-1α (scale bar: 100 µm, magnification 10X). *Right graph*, quantification of cumulative sprout length (µM). Bars are means ±SD (*p < 0.0002 vs. CTR; **p < 0.0002 vs. HUVEC CM- or fibroblast CM-treated cells; *n* = 3).

To further uncover the role of ET-1 in HG-SOC/stroma interactions, we explored whether the ET-1-driven signaling machinery was required for the rewiring of fibroblast functions. In particular, in human fibroblasts expressing both ET_A_R and ET_B_R (Fig. 5A), ET-1 stimulation favored the induction of activated fibroblast markers, such as α-smooth muscle actin (α-SMA), vimentin and fibronectin. This effect was reduced upon treatment with macitentan (Fig. 5B), indicating that the ET-1/ET-1R axis promotes fibroblast activation [50]. The ET-1 stimulation mediated the downregulation of YAP dephosphorylation (Supplementary Fig. 5A) and favored YAP and HIF-1α nuclear accumulation and activation (Fig. 5C), which were both prevented by macitentan treatment. In addition, PLA analysis revealed the existence of YAP/HIF-1α nuclear complexes in ET-1-treated fibroblasts, which were dampened by macitentan (Fig. 5D). Co-IP analysis confirmed that ET-1 may drive the YAP/HIF-1α physical interaction. Remarkably, the nuclear complex included p53, which often displays altered functionality in the transcriptional activity of activated fibroblasts [51, 52] (Fig. 5E). The analysis of fibroblast CM revealed that ET-1R activation enhanced the release of VEGF and ET-1, an effect prevented by macitentan, as well as by YAP or HIF-1α silencing (Fig. 5F, G and Supplementary Fig. 5B, C).

Additionally, we observed that, similar to the effect produced by exogenous stimulation with ET-1, fibroblasts treated with HG-SOC CM and HG-SOC cells treated with fibroblast CM both displayed a greater migration rate than untreated cells (Supplementary Fig. 5E, F). Conversely, treatment with macitentan or CM previously depleted for YAP, HIF-1α or p53 expression reduced fibroblast and tumor cell migratory potential (Supplementary Fig. 5B–F).

Similarly, in HG-SOC/fibroblast co-cultures, ET-1-stimulated cells moved into the area of the cell free gap, resulting in a gap closure of approximately 67%, similar to the effect of HG-SOC and fibroblast CM (gap closure of 66 and 64%, respectively; Fig. 5H). Notably, treatment with either macitentan or depletion of YAP, HIF-1α or p53 proteins suppressed cell migration (Fig. 5H). In addition, in 3D PD HG-SOC spheroids, stimulation with ET-1, EC or fibroblast CM significantly enhanced the formation of invasive sprouts in the surrounding matrix. This effect was impaired by treatment with macitentan, as well as by YAP or HIF-1α silencing (Fig. 5I). Collectively, these findings suggest that ET-1-mediated reciprocal YAP/HIF-1α/mutp53 node signal activation in neighboring cells favors invasive behavior and metastatic dissemination.

### Macitentan, by disrupting the cooperative YAP/HIF-1α/mutp53 adaptive signaling, enhances HG-SOC sensitivity to olaparib

**Fig. 6:**
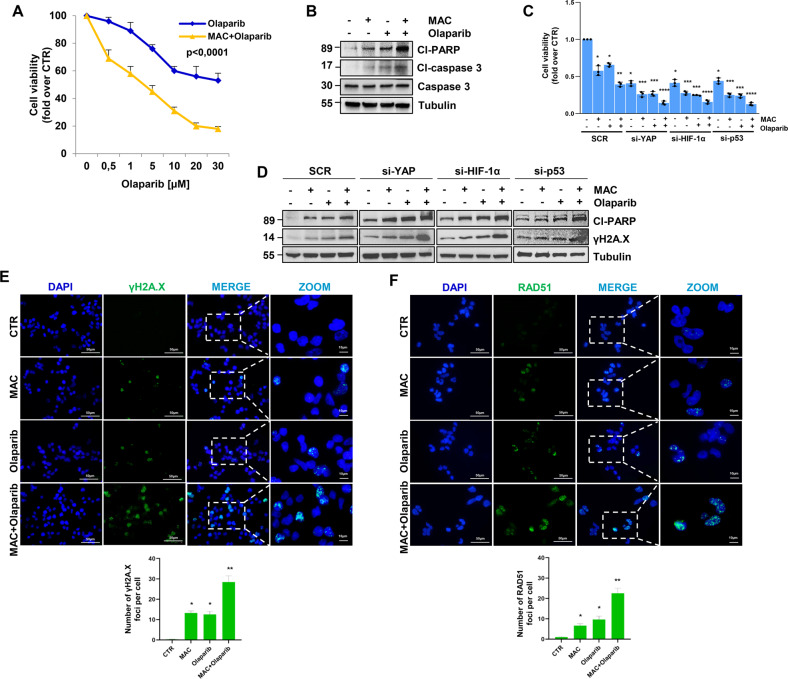
ET-1R blockade by macitentan, hampering the mutp53/YAP/HIF-1α axis, enhances HG-SOC cell sensitivity to olaparib, promoting DNA damage and apoptosis. **A** Effect of different concentrations of olaparib, alone or in combination with MAC (1 µM) for 48 h, on PMOV10 cell vitality. Data points are means ± SD (*p < 0.0001 vs. olaparib; *n* = 3). **B** IB analysis of cleaved-PARP (cl-PARP), caspase 3 and cleaved-caspase 3 (cl-caspase 3) expression in PMOV10 cells treated with MAC and/or olaparib (1 µM). Tubulin represents the loading control. **C** Effect of treatment with MAC and/or olaparib, alone or in combination for 48 h, on the vitality of PMOV10 cells transfected with SCR, si-YAP, si-HIF-1α or si-p53 for 72 h. Bars are the means ± SD (*p < 0.0005 vs. SCR CTR; **p < 0.002 vs. SCR olaparib; ***p < 0.002 vs. SCR olaparib or SCR MAC; ****p < 0.0006 vs. SCR MAC + olaparib; *n* = 3). **D** IB analysis of cleaved-PARP and phospho-Histone H2A.X (S139; γH2A.X) expression in PMOV10 cells treated as in C. Tubulin represents the loading control. Representative images of blots of 3 independent experiments are shown in **B** and **D**. **E**, **F** Representative images of γH2A.X (**E**) and RAD51 (**F**) foci (green) evaluated by IF in PMOV10 cells stimulated with MAC and/or olaparib for 24 h (scale bar: 50 µm, magnification 64X). Nuclei are stained blue (DAPI). Images are magnified to show the γH2A.X (**E**) and RAD51 (**F**) foci. The bottom graphs represent the quantification of the number of γH2A.X and RAD51 foci. Bars are means ± SD (*p < 0.005 vs. CTR; **p < 0.005 vs. olaparib*; n* = 3).

Given that YAP, which is downstream of ET-1R, is emerging as a modulator of the response to different anticancer therapies [12, 13, 20–22], we explored whether the signal network between mutp53/YAP and HIF-1α instigates an escape strategy from DNA damaging agents, such as the PARPi olaparib. Interestingly, treatment with macitentan increased sensitivity to olaparib (Fig. 6A and Supplementary Fig. 6A) and the levels of cleaved-PARP and cleaved-caspase 3 (cl-PARP and cl-caspase 3), well-known markers of cell apoptosis in HG-SOC cells (Fig. 6B and Supplementary Fig. 6B, C). Of relevance, we confirmed the sensitization effect of macitentan in a breast cancer cell line MDA-MB-468, expressing both ET_A_R and ET_B_R [12], as shown by the increased PARP cleavage and enhanced expression of γH2A.X, upon the combination treatment of macitentan and olaparib (Supplementary Fig. 6D), indicating that ET-1R antagonists may be combined with PARPi in various ET_A_R-expressing tumor types.

Next, we screened the effect of selective ET_A_R antagonists (zibotentan and BQ123) and selective ET_B_R antagonist (BQ788) compared to macitentan. Macitentan, zibotentan or BQ123, but not BQ788, enhanced cl-PARP, cl-caspase 3 and γH2A.X expression, suggesting an ET_A_R-specific mechanism of regulation of pro-apoptotic and DNA damaging markers (Supplementary Fig. 6E). Of note, macitentan was more effective than zibotentan in enhancing HG-SOC sensitivity to olaparib (Supplementary Fig. 6F).

**Fig. 7:**
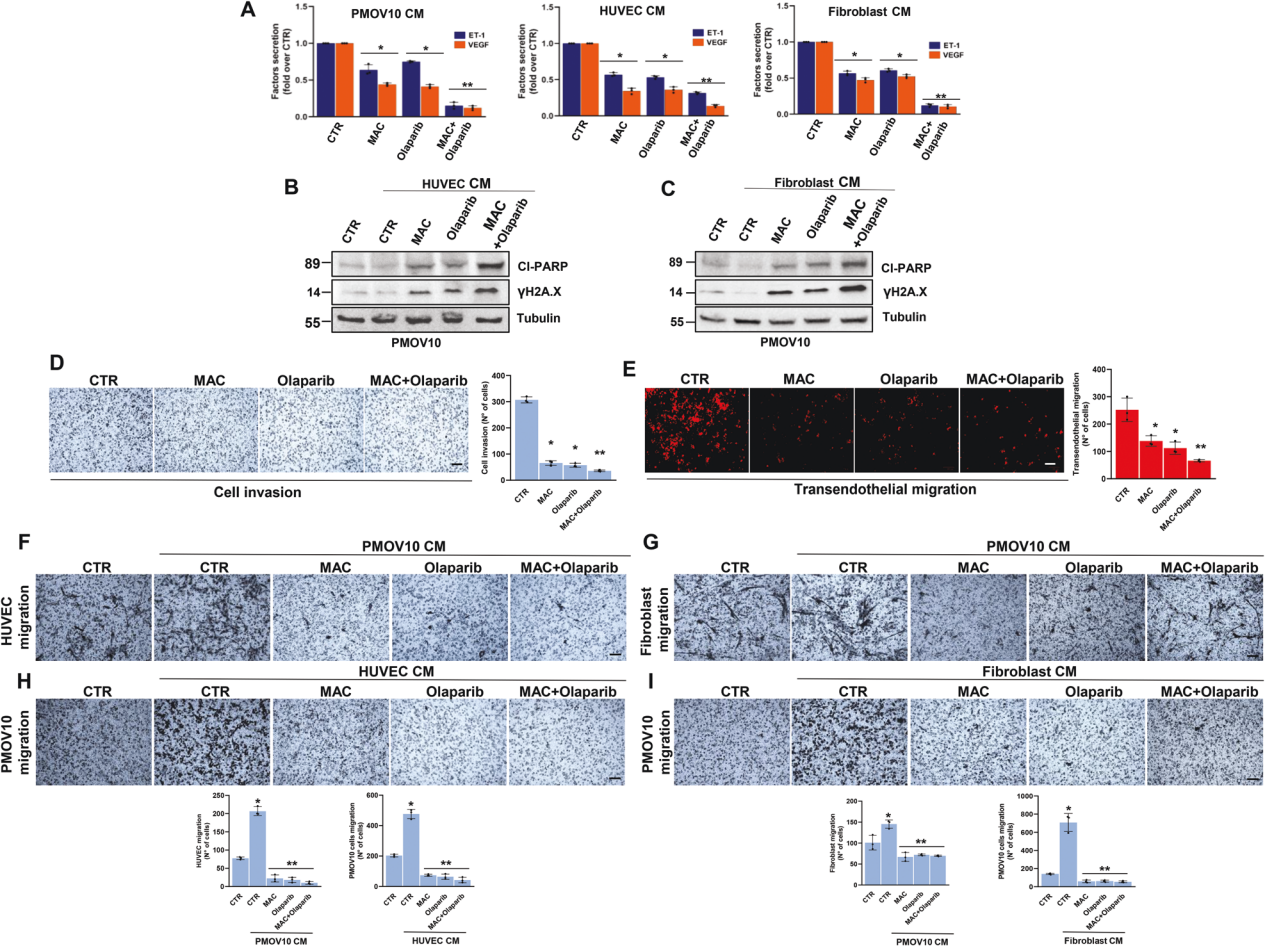
The combination of macitentan and olaparib, impairing the p53/YAP/HIF-1α-mediated release of ET-1 and VEGF, interferes with tumor/stroma signal reciprocity and the pro-invasive behavior of HG-SOC cells. **A** ELISA for ET-1 and VEGF released by PMOV10 cells, HUVEC and fibroblasts treated with macitentan or olaparib, alone or in combination for 24 h. Bars are the means ± SD (*p < 0,002 vs. CTR; **p < 0,0006 vs. olaparib; *n* = 3). **B**, **C** IB analysis of cl-PARP and γH2A.X expression in PMOV10 cells treated with CM from HUVEC (**B**) or fibroblasts (**C**) prestimulated with MAC or olaparib, alone or in combination for 24 h. Tubulin represents the loading control. Representative images of blots of 3 independent experiments are shown. **D** Invasion assay of PMOV10 cells treated with MAC or olaparib, alone or in combination for 24 h. Representative images of the invading cells were photographed (scale bar: 100 µm, magnification 20X) (*left panels*) or counted (*right graph*). Bars are the means ± SD (*p < 0.0002 vs. CTR; **p < 0.02 vs. olaparib-treated cells; *n* = 3). **E** Transendothelial migration assay of PMOV10 cells treated with MAC or olaparib, alone or in combination for 24 h. Representative images of the transmigrated cells were photographed (scale bar: 100 µm, magnification 20X) (*left panels*) or counted (*right graph*). Bars are means ± SD (*p < 0.0002 vs. CTR; **p < 0.0003 vs. olaparib-treated cells; *n* = 3). **F**, **G** Migration assay of HUVEC (F) or fibroblasts (G) treated with CM from PMOV10 cells prestimulated with MAC or olaparib, alone or in combination for 24 h. Representative images of migrating cells were photographed (scale bar: 100 µm, magnification 20X) (*upper panels*) or counted (*bottom graphs*). Bars are means ± SD (*p < 0.02 vs. CTR; **p < 0.0008 vs. PMOV10 CM treated cells; *n* = 3). **H**, **I** Migration assay of PMOV10 cells treated with CM from HUVEC (H) or fibroblasts (I) prestimulated with MAC or olaparib, alone or in combination for 24 h. Representative images of migrating cells were photographed (scale bar: 100 µm, magnification 20X) (*upper panels*) or counted (*bottom graphs*). Bars are means ± SD (*p < 0.0007 vs. CTR; **p < 0.0005 vs. HUVEC or fibroblast CM-treated cells; *n* = 3).

Intriguingly, HG-SOC cells depleted for YAP, HIF-1α or p53 expression and concomitantly treated with either macitentan or olaparib, were less viable than the control cells (Fig. 6C). In line with these observations, we detected increased apoptosis, as shown by the increased protein levels of cl-PARP and γH2A.X, in HG-SOC cells depleted of YAP, HIF-1α or p53 and co-treated with macitentan and olaparib (Fig. 6D and Supplementary Fig. 6G, H). The induction of severe DNA damage was evidenced by the increased number of γH2A.X and RAD51 foci, markers for double-stranded DNA breaks, in HG-SOC cells treated with a combination of macitentan and olaparib (Fig. 6E, F). Remarkably, such combinatorial treatment, constraining the activity of ET-1-driven p53/YAP/HIF-1α machinery contextually in tumor cells, EC and activated fibroblasts, induced a drastic reduction in the release of ET-1 and VEGF (Fig. 7A), and inhibited the compensatory signals that contributed to the PARPi escape pathway. Indeed, macitentan rendered HG-SOC cells more sensitive to olaparib, as shown by the increase of cl-PARP and γH2A.X levels, even in HG-SOC cells treated with CM from HUVEC or activated fibroblasts (Fig. 7B, C) and inhibited invasive behavior and transendothelial migration (Fig. 7D, E). Moreover, the combination of macitentan with olaparib interfered with the bidirectional signal exchange between HG-SOC cells and EC (Fig. 7F, H), as well as HG-SOC cells and activated fibroblasts (Fig. 7G, I), thus inhibiting tumor cell migration (Fig. 7H, I). Overall, these findings indicate that macitentan plus olaparib interrupts tumor/stroma communication, rendering HG-SOC cells more sensitive to olaparib.

### Macitentan inhibits metastatic dissemination and enhances olaparib efficacy in HG-SOC patient-derived xenografts

**Fig. 8:**
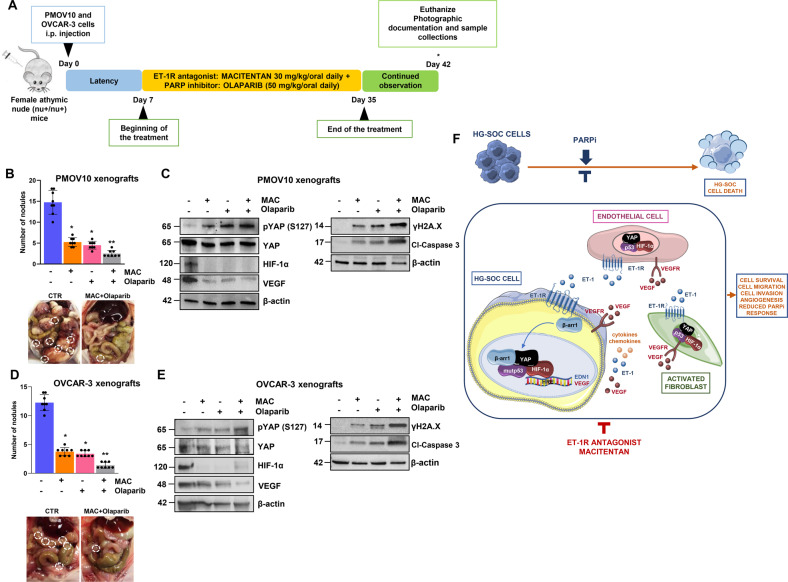
Macitentan reduces HG-SOC metastatic potential and sensitizes to olaparib in vivo. **A** Treatment schedule of patient-derived HG-SOC xenografts (PDX) and OVCAR-3 xenografts. **B**, **D** The number of tumor nodules examined at the end of the treatment. Bars are the means ± SD (*p < 0.0002 vs. vehicle-treated mice (CTR); **p < 0.0004 vs. olaparib-treated mice; *n* = 2). *Right panels*, Representative i.p. The metastatic nodules are indicated by white dotted-line circles. **C**, **E** pYAP (S127), YAP, HIF**-**1α, VEGF, γH2A.X and cl-caspase 3 protein expression in total cell lysates of i.p. nodules was evaluated by IB analysis. β-actin represents the loading control. Representative images of blots of 2 independent experiments are shown. **F** Working model illustrating how under the guidance of the ET-1/ET-1R axis, mutp53 anchors YAP and HIF-1α to DNA, turning on a cooperative transcriptional program in HG-SOC cells, endothelial cells and activated fibroblasts that culminates with the release of soluble mediators, such as ET-1, and how the amplification of a self-feeding circuit blunts the effect of PARPi. ET-1R blockade, dismantling the cross-talk between HG-SOC, EC, and activated fibroblasts, empowers olaparib efficacy, representing a valid companion for PARPi-based therapy.

Next, we evaluated the therapeutic efficacy of the pharmacological inhibition of ET-1R by macitentan in potentiating the effect of olaparib by controlling the in vivo metastatic dissemination of HG-SOC patient-derived xenografts (PDX). We assessed the effects of the following treatments in HG-SOC PDX and OVCAR-3 xenografts: control (vehicle) versus macitentan (30 mg/kg/oral daily) and/or olaparib (50 mg/kg/oral daily), in monotherapy or in combination therapy (Fig. 8A). The drug combination was well tolerated with no weight loss evidenced in all treatment groups. The combination significantly reduced the number and the size of tumor nodules. The effect was synergistic, as evaluated by the Chou–Talalay method [41], in HG-SOC PDX (CDI 0.09) and OVCAR-3 xenografts (CDI 0.1) treated with the combinatorial treatment schedule of macitentan and olaparib (Fig. 8B, D). In parallel, immunoblotting analysis performed with protein extracts isolated from tumor nodules revealed a significant increase in pYAP protein levels upon combination therapy. Notably, combinatorial treatments demonstrated the ability to reduce HIF-1α and VEGF protein expression, and in parallel to increase cl-caspase 3 and γH2A.X expression levels (Fig. 8C, E). These findings provide strong in vivo evidence of how macitentan, by simultaneously suppressing the intertwined YAP and HIF-1α signaling pathways, sensitizes HG-SOC PDX to olaparib, demonstrating a greater anti-metastatic and apoptotic effects, when administered as a combinatorial treatment regimen.

## Discussion

The cross-talk between HG-SOC cells and the TME can also result in self-enhancing loops that contribute to generating poor responses to the available therapies and to the dismal prognosis of HG-SOC patients [1, 53]. Beyond immune cells, EC and activated fibroblasts work in concert with the ECM and other secreted molecules, such as growth factors, cytokines, and chemokines [5–8], to establish an environment that supports the survival of cancer. In this regard, the identification of the molecular critical nodes that integrate TME components may pave the road to more effective therapeutic strategies. In an effort to deeply understand the treatment-escaping mechanisms that are able to model HG-SOC/TME intercommunication, we documented how the ET-1/ET-1R circuit, by mimicking the effects of hypoxia, drives the p53/YAP/HIF-1α physical and functional alliance in the tumoral context and concurrently in EC and activated fibroblasts, to chart an interconnected route. The formation of this communication hub, by connecting HG-SOC with the surrounding stroma, represents an alternative pathway to survive to PARPi-based therapy. The current understanding of the mechanisms underlying the therapeutic efficacy of PARPi is still evolving and is mainly focused on tumor cell-intrinsic mechanisms. Our findings provide insights into the role of stromal elements in PARPi therapy and demonstrate that ET-1R-guided bidirectional interactions between tumor and stromal cells regulate PARPi response (Fig. 8F). In particular, we discovered that ET-1R, by driving the nuclear accumulation of p53, YAP and HIF-1α simultaneously in HG-SOC cells, EC and activated fibroblasts, dictates the formation and activation of a transcriptional apparatus. This leads to the enhanced release of a wide array of molecules, including ET-1 and VEGF circuits, thereby fueling a mechanism of PARPi evasion. Our findings support and add greater relevance to previous studies demonstrating that, in normoxic conditions, but even more so in hypoxic conditions, the alliance between HIF-1α and mutp53 signals potentiates the release of soluble factors that remodel the ECM and affect stromal cell activity, thereby enhancing tumor growth and metastatic potential in breast cancer [36, 37]. Given that the tumor-promoting TME phenotype is mediated via diverse mechanisms, we identified the ET-1R-mediated p53/YAP/HIF-1α hub as a therapeutic vulnerability that impacts the secretome of HG-SOC cells and the crosstalk between tumor cells with EC and activated fibroblasts, thus regulating the DNA damage response and the efficacy of PARPi treatment.

While *TP53* mutations are dominant in HG-SOC cells, it is now commonly accepted that stromal elements, including EC and activated fibroblasts, do not harbor genetic alterations in *TP53*. However, the canonical p53 functionality in such stromal components is frequently compromised, switching from tumor-suppressive to tumor-supportive, rendering these cells gradually more aggressive [45, 51, 52]. Therefore, p53’s ability to interact with critical partners, such as HIF-1α and YAP, in EC and activated fibroblasts, represents one possibility of how this complex generates a tumor-supportive stromal compartment. In parallel to the autocrine/paracrine signaling modality of different proinvasive and angiogenic factors and inflammatory cytokines and chemokines, the formation of the p53/YAP/HIF-1α communication node is of particular interest because its activation depends on the ET-1 signal route, and in a self-magnifying circuit, in both tumor cells and stromal elements, this ultimately sustains escape from PARPi and unfavorable outcomes. Interestingly, the dynamic multifactorial connection induced by ET-1 between EC, activated fibroblasts and HG-SOC cells includes VEGF, suggesting that ET-1 and VEGF communication cues may affect pro-malignant properties and PARPi sensitivity. Indeed, the integration of ET-1 and VEGF circuits allows HG-SOC cells to escape drug control by maintaining major pro-survival signals in a continuous “on” state. Here, we show that a dual ET-1R antagonist not only promotes antitumor effects on HG-SOC cells, but also interrupts these feed-forward loops in the TME, thereby halting HG-SOC progression efficiently and rendering tumors more susceptible to PARPi therapy. Indeed, PARPi in combination with macitentan demonstrates synergistic antitumor efficacy compared with PARPi in PD HG-SOC samples and in orthotopic PDX, thereby emerging as a rational therapeutic option in combination with PARPi for HG-SOC patients. Our results are aligned with recent findings that emphasize how the repurposing of macitentan simultaneously interferes with tumor cell intrinsic and extrinsic conditions by regulating the function of cancer-associated fibroblasts and tumor-associated EC in different tumor models [53, 54]. In line with these observations, it has been recently reported that ET_A_R is one of the critical elements of the complex machinery for extracellular vesicle biogenesis and secretion, acting as an important mediator of cell-cell communications [55]. Moreover, ET_A_R expression has been associated with anti-PD-1 therapy resistance in breast cancer [56], as well as macitentan, which prevents tumor-mediated immune evasion and improves immune checkpoint blockade [56, 57]. This finding supports the idea that macitentan could be combined with other drugs, potentially targeting the TME, for the benefit of ET_A_R-overexpressing cancer patients.

Remarkably, the high YAP/ET_A_R/HIF-1α gene expression is a predictive signature of poor prognosis, negatively impacting the overall and relapse-free survival of HG-SOC patients. These findings further support the notion that the ET-1R-guided mutp53/YAP/HIF-1α transcriptional alliance critically contributes to the worst HG-SOC disease outcomes.

Overall, our mechanistic and preclinical study provides an improved therapeutic option for HG-SOC patients harboring *TP53* mutations with a low response to olaparib, especially those with high ET_A_R/YAP/HIF-1α expression. We have shown that the combination of macitentan and olaparib leads to increased DNA damage and metastatic nodule inhibition, providing data that may be applied to the clinical setting. The emerging approach for cancer treatment of macitentan may be a tumor/stroma communication blockade, indicating that the ET-1R antagonist can be combined with olaparib to resensitize tumors to PARPi therapy and potentially improve HG-SOC patient outcome.

## Supplementary information

Supplementary Figures

Supplementary Information

Original Data File

Reproducibility checklist

## Data Availability

All data generated and analysed during the current study are included in this article and its supplementary information file or from the corresponding authors (A.B or P.T) on reasonable request. The uncropped images of the blots, can be found in the original western blots file. PMOV10 HG-SOC cells will be made available to academic researchers with material transfer agreement. Further information on research design is available in the Reproducibility checklist. GSEA: Public available data used in this article were obtained from: GSEA Gene Set Enrichment Analysis (https://www.gsea-msigdb.org/gsea/index.jsp).

## Code availability

The RNA-seq data have been deposited at the GEO repository database with the accession code GSE196065.
