# Supplementary material for: Targeting tumor-stroma communication by blocking endothelin-1 receptors sensitizes high-grade serous ovarian cancer to PARP inhibition
Source: Cell Death Dis. 2023 Jan 5;14(1):5. doi: 10.1038/s41419-022-05538-6 (PMC9816119; doi:10.1038/s41419-022-05538-6)
Supplement: Supplementary file 1 — Supplementary Figures [file 41419_2022_5538_MOESM1_ESM.pdf]

Supplementary Figures

Targeting tumor-stroma communication by blocking endothelin-1 receptors sensitizes high-grade serous ovarian cancer to PARP inhibition

Piera Tocci[1*], Celia Roman[1], Rosanna Sestito[1], Valeriana Di Castro[1], Andrea Sacconi[2], Ivan Molineris[3], Francesca Paolini[4], Mariantonia Carosi[5], Giovanni Tonon[6,7], Giovanni Blandino[2] and Anna Bagnato[1*]

[1]Preclinical Models and New Therapeutic Agents Unit, Istituto di Ricovero e Cura a Carattere Scientifico (IRCCS), Regina Elena National Cancer Institute, Rome, Italy; [2]Translational Oncology Research Unit, IRCCS, Regina Elena National Cancer Institute, Rome, Italy; [3]Department of Life Science and System Biology, University of Turin, Turin, Italy; [4]Tumor Immunology and Immunotherapy Unit, IRCCS, Regina Elena National Cancer Institute, Rome, Italy; [5] Pathology Unit, Department of Research, Diagnosis and Innovative Technologies, IRCCS, Regina Elena National Cancer Institute, Rome, Italy; [6]Center for Omics Sciences (COSR) and Functional Genomics of Cancer Unit, Division of Experimental Oncology, IRCCS San Raffaele Scientific Institute, Milan, Italy; [7]Università Vita-Salute San Raffaele, 20132, Milan, Italy.

*****Correspondence and requests for materials should be addressed to Anna Bagnato (email: annateresa.bagnato@ifo.it) or to Piera Tocci (email: piera.tocci@ifo.it)

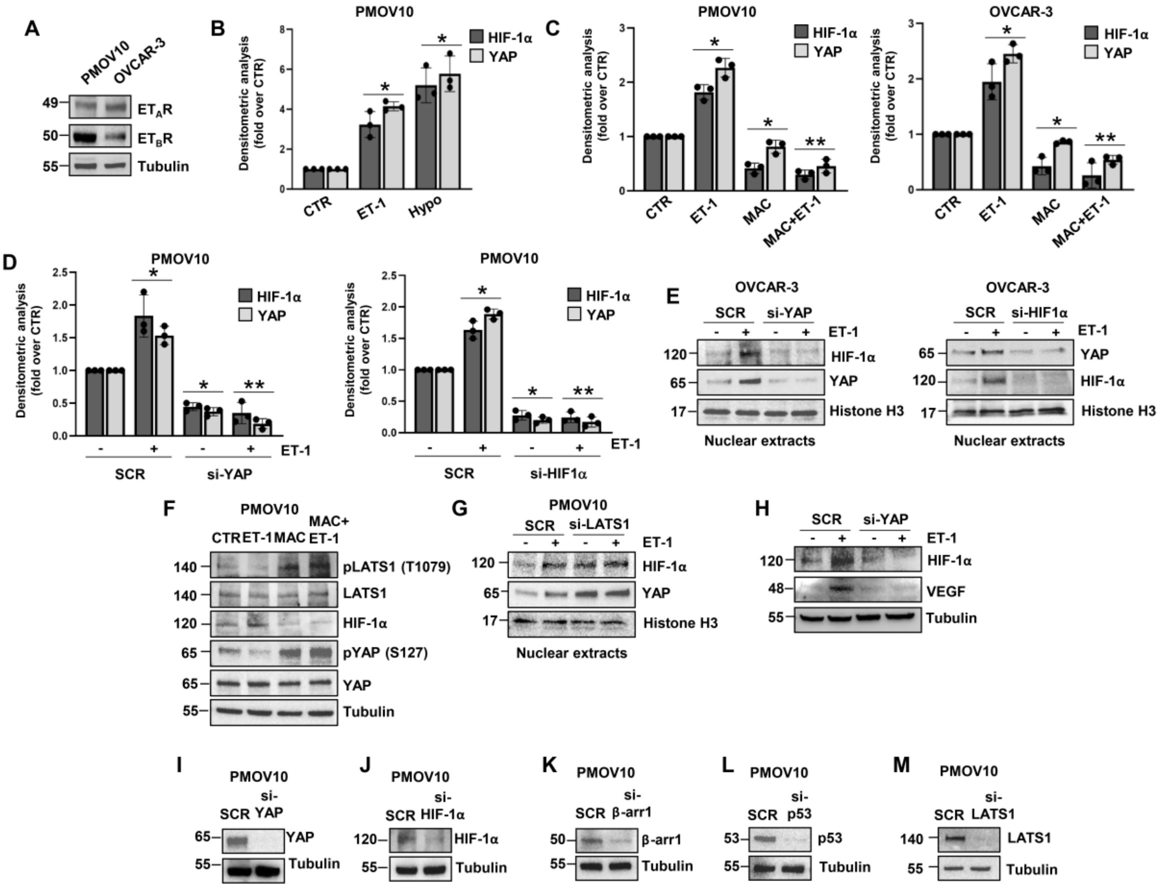

Fig. S1. PMOV10 and OVCAR-3 express the ET-1R. A. Immunoblotting (IB) analysis for ET_AR and ET_BR protein expression in patient-derived HG-SOC cells (PMOV10) and HG-SOC cell line OVCAR-3. Tubulin was used as loading control. Representative images of blots of 3 independent experiments are shown. B. Quantification of HIF-1α and YAP protein expression in nuclear extracts of PMOV10 cells normalized to Histone H3 of IB analyses shown in Figure 1D. Values are the mean ±SD expressed as fold induction (*, p<0.004 vs. CTR; *n=3*). C. Quantification of HIF-1α and YAP protein expression in nuclear extracts of PMOV10 (*left*) and OVCAR-3 (*righ*) cells normalized to Histone H3 of IB analyses shown in Figure 1E and F. Values are the mean ±SD expressed as fold induction (*, p<0.02 vs. CTR; **, p<0.002 vs. ET-1; *n=3*). D. Quantification of HIF-1α and YAP protein expression in nuclear extracts of PMOV10 cells silenced for YAP (*left*) or HIF-1α (*right*) normalized to histone H3 of IB analyses shown in Figure 1G. Values are the mean ±SD expressed as fold induction (*, p<0.01 vs. CTR; **, p<0.002 vs. ET-1;*n=3*). E. IB analyses for HIF-1α and YAP

protein expression in nuclear extracts of OVCAR-3 cells stimulated with ET-1 for 2 h and 72 h transfected with SCR, si-YAP (*left*) or si-HIF-1α (*right*). Histone H3 represents the loading control. F. Expression of pLATS1 (T1079), LATS1, HIF-1α, pYAP (S127) and YAP proteins analyzed by IB in total extracts of PMOV10 cells stimulated with ET-1 and/or macitentan (MAC) for 2 h. Tubulin represents the loading control. G. IB analysis for HIF-1α and YAP nuclear expression in PMOV10 cells 72 h silenced for LATS1 and stimulated or not with ET-1 for 2 h. Histone H3 represents the loading control. H. Total extracts of PMOV10 cells with or without YAP silencing for 72 h and stimulated or not stimulated with ET-1 for 48 h were subjected to IB for HIF-1α and VEGF. Tubulin represents the loading control. I-M. YAP (I), HIF-1α (J), β-arrestin1 (β-arr1) (K), p53 (L), and LATS1 (M) protein expression in PMOV10 cells silenced or not for YAP (I), HIF-1α (J), β-arr1 (K), p53 (L), or LATS1 (M) for 72 h. Tubulin represents the loading control. Representative images of blots of 3 independent experiments are shown in E-M.

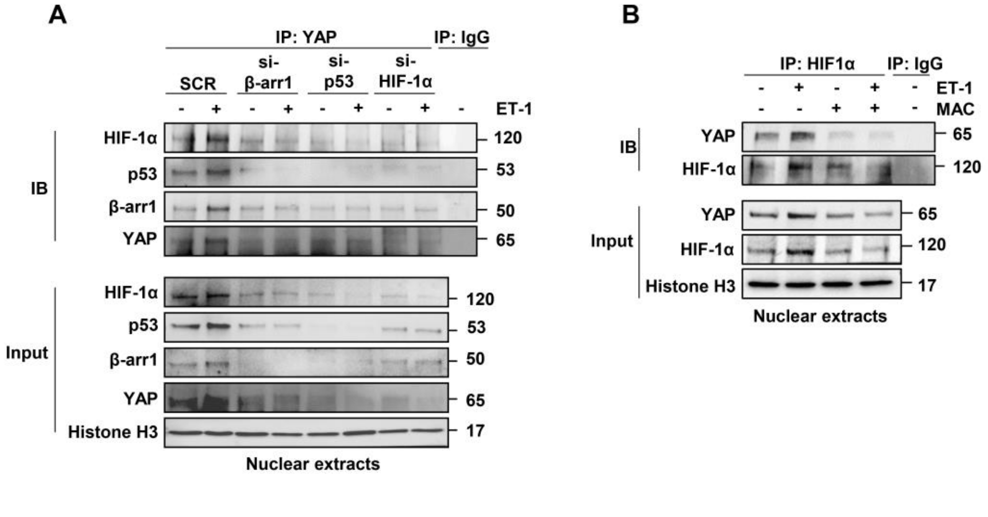

Fig. S2. ET-1 guides the formation of the multimeric mutp53/YAP/HIF-1α/β-arr1 nuclear complex. A. Nuclear extracts of PMOV10 cells silenced for β-arr1, p53 and HIF-1α for 72 h and stimulated with ET-1 for 2 h were IP for YAP using anti-YAP, or anti-IgG and IB using anti-YAP, anti-HIF-1α, anti-p53, and anti-β-arr1 antibodies (Abs). Histone H3 represents the loading control.

B. Nuclear extracts of PMOV10 cells stimulated with ET-1 and/or MAC for 2 h were IP for HIF-1α using anti-HIF-1α, or anti-IgG and IB using anti-YAP or anti-HIF-1α Abs. Histone H3 represents the loading control. Representative images of blots of 3 independent experiments are shown.

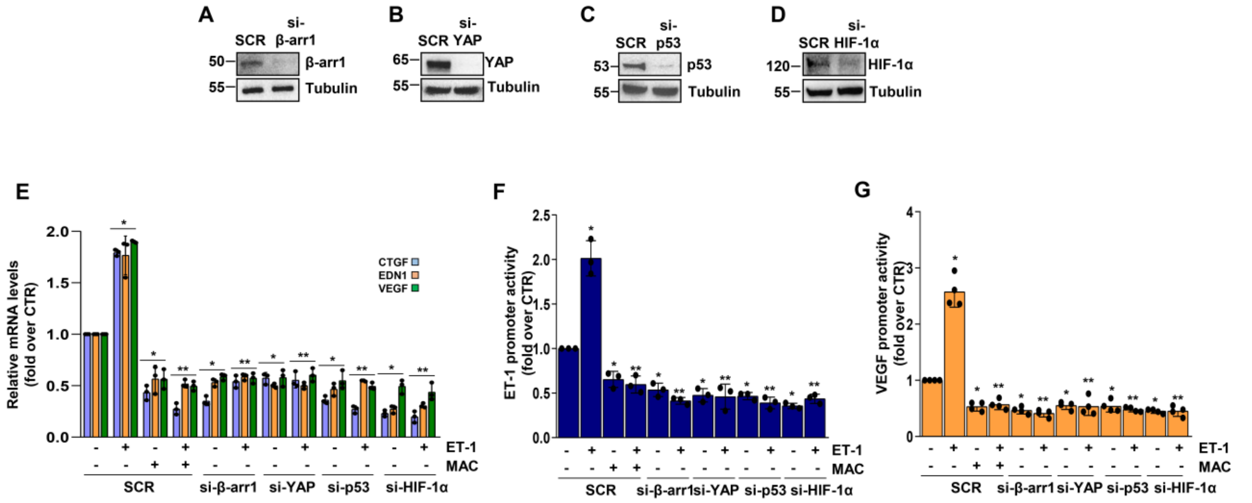

Fig. S3. mutp53/YAP mediate the ET-1R-induced HIF-1α transcriptional activity sustaining HG-SOC cell invasion and transendothelial migration. A-D. β-arrestin1 (A), YAP (B), p53 (C) and HIF-1α (D) protein expression in OVCAR-3 cells silenced or not for β-arrestin1 (A), YAP (B), p53 (C) and HIF-1α (D) for 72 h. Tubulin represents the loading control. Representative images of blots of 3 independent experiments are shown in A-D. E. Expression analysis (qRT-PCR) of the mRNA levels of YAP/HIF-1α target genes, *CTGF*, *EDN1* and *VEGF*, in OVCAR-3 cells stimulated with ET-1 and treated with MAC for 24 h or silenced for β-arr1, YAP, p53 and HIF-1α for 72 h. Bars are means ± SD (*, $p<0.005$ vs. CTR; **, $p<0.0005$ vs. ET-1; $n=3$). F, G. ET-1 promoter activity (F) and VEGF promoter activity (G) measured in OVCAR-3 cells silenced as in E, co-transfected with ET-1 promoter-luc construct (F) or VEGF promoter-luc construct (G) for 24 h, and stimulated with ET-1 and/or MAC for 24 h. Bars are means ± SD (*, $p<0.003$ vs. CTR; **, $p<0.0005$ vs. ET-1; $n=3$).

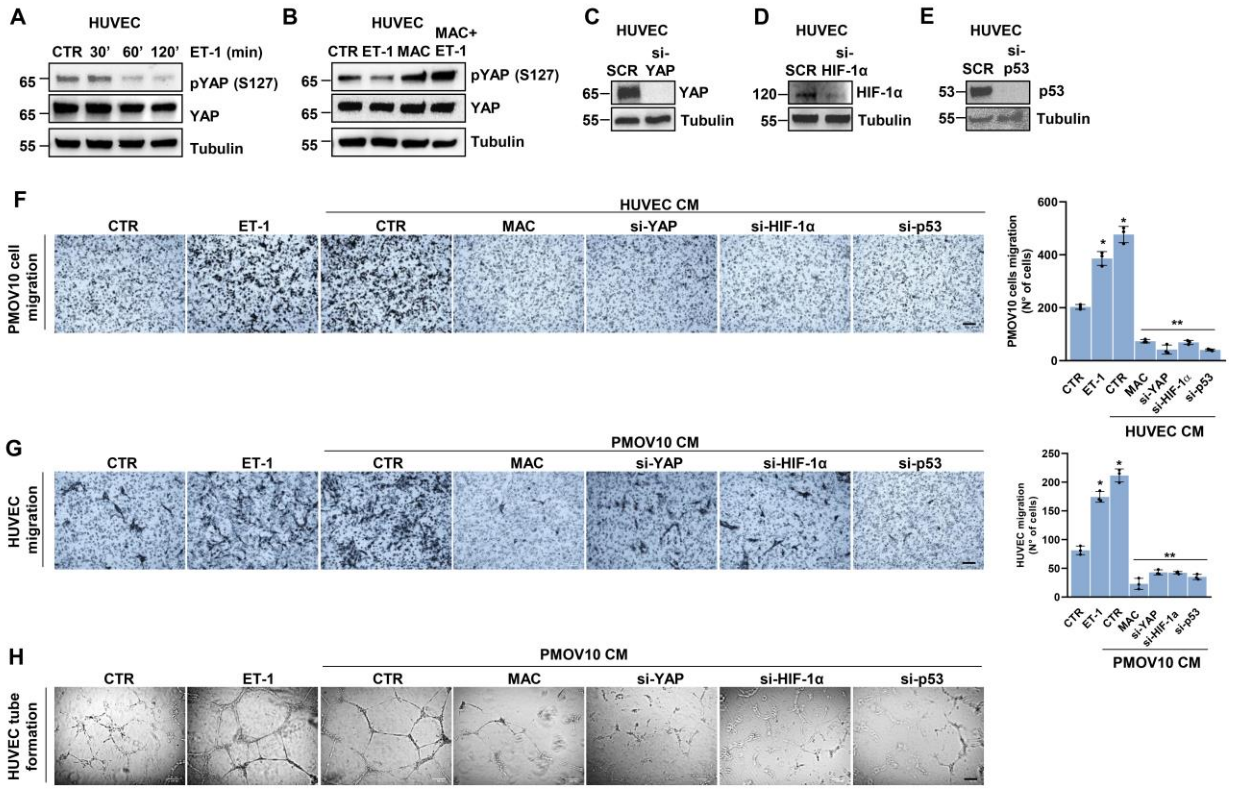

Fig. S4. ET-1 mediates the HG-SOC/endothelial cells cross-talk through the p53/YAP/HIF-1α signaling. A. IB analysis for pYAP (S127) and YAP protein expression in HUVECs stimulated with ET-1 for the indicated times. Tubulin represents the loading control. B. IB analysis for pYAP (S127) and YAP protein expression in total extracts of HUVECs stimulated with ET-1 and/or MAC for 2 h. C-E. HUVECs silenced or not for YAP (C), HIF-1α (D) or p53 (E) for 72 h were IB for YAP (C), HIF-1α (D) and p53 (E). Representative images of blots of 3 independent experiments are shown in A-E. F. Migration assay of PMOV10 cells stimulated or not with ET-1 or with conditioned media (CM) from HUVECs treated or not with MAC for 24 h or silenced for YAP, HIF-1α or p53 for 72 h. Representative images of the migrating cells were photographed (scale bar: 100μm, magnification 20X) (*left panels*) or counted (*right graph*). Bars are means ± SD (* $p<0.0004$ vs. CTR; ** $p<0.0002$ vs. HUVEC CM treated cells; *n=3*). G. Migration assay of HUVECs stimulated or not with ET-1, or with CM from PMOV10 cells treated or not with MAC for 24 h, or silenced for YAP, HIF-1α or p53 for 72 h. Representative images of the migrating cells were photographed (scale bar: 100μm,

magnification 20X) (*left panels*) or counted (*right graph*). Bars are means ± SD (* p<0.0004 vs. CTR; ** p<0.0002 vs. PMOV10 CM treated cells; *n=3*). H. Tube formation assay of HUVECs stimulated as in G. Representative images of the tubes were photographed (scale bar: 100µm, magnification 20X).

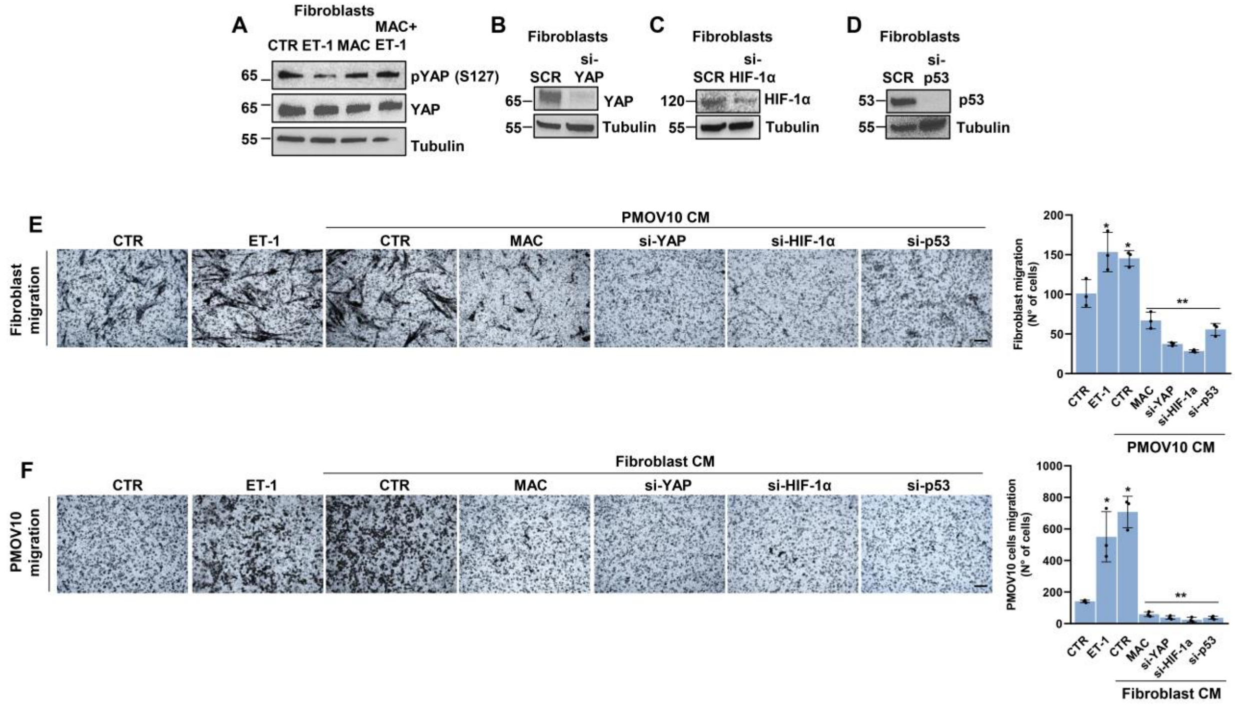

Fig. S5. ET-1 mediates the HG-SOC/fibroblasts cross-talk through the p53/YAP/HIF-1α signaling. A. IB analysis for pYAP (S127) and YAP protein expression in total extracts of fibroblasts stimulated with ET-1 and/or MAC for 2 h. Tubulin represents the loading control. Representative images of blots of 3 independent experiments are shown. B-D. fibroblasts silenced or not for YAP (B), HIF-1α (C) or p53 (D) for 72 h were IB with anti-YAP (B), anti-HIF-1α (C), or anti-p53 (D) abs. Tubulin represents the loading control. Representative images of blots of 3 independent experiments are shown in A-D. E. Migration assay of fibroblasts stimulated or not with ET-1 or with CM from PMOV10 cells treated or not with MAC for 24 h or silenced for YAP, HIF-1α or p53 for 72 h. Representative images of the migrating cells were photographed (scale bar: 100µm, magnification 20X) (*left panels*) or counted (*right graph*). Bars are means ± SD (* p<0.05 vs. CTR; ** p<0.0008

vs. PMOV10 CM treated cells; *n=3*). F. Migration assay of PMOV10 cells stimulated or not with ET-1 or with CM from fibroblasts treated or not with MAC for 24 h or silenced for YAP, HIF-1α or p53 for 72 h. Representative images of the migrating cells were photographed (scale bar: 100μm, magnification 20X) (*left panels*) or counted (*right graph*). Bars are means ± SD (* p<0.02 vs. CTR; **p<0.0005 vs. fibroblast CM treated cells; *n=3*).

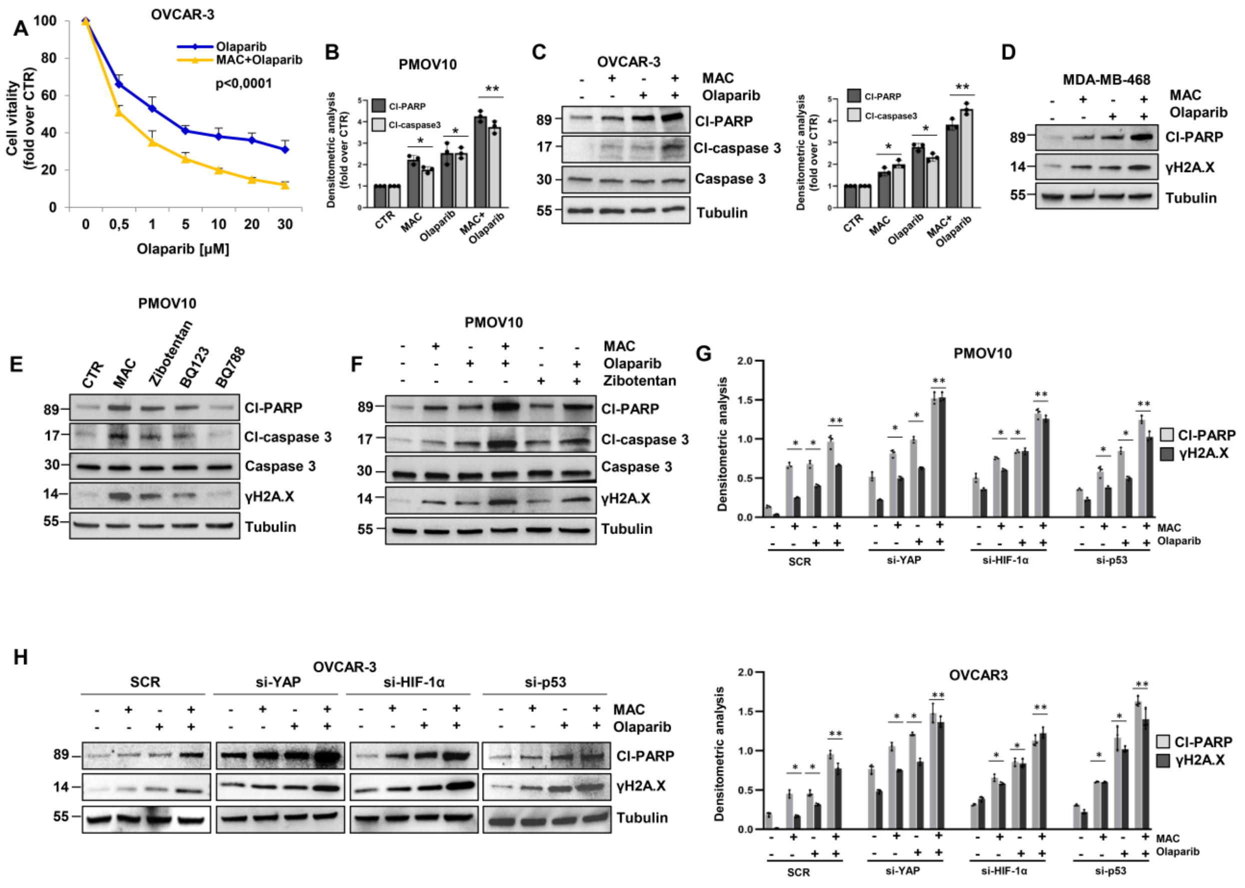

Fig. S6. Macitentan, interfering with mutp53/YAP/HIF-1α oncogenic network, enhances HG-SOC cells sensitivity to olaparib inducing DNA damage and apoptosis. A. Effect of exposure to different concentrations of olaparib (from 0,5 to 30 μM) alone or in combination with MAC (1μM) after 48 h on cell vitality of OVCAR-3. Data points are means ± SD (*, p<0.0001 vs. olaparib; n=3). B. Quantification of cl-PARP and cl-caspase 3 total extracts of PMOV10 cells treated with MAC or olaparib (20 μM), alone or in combination for 48 h normalized to tubulin of IB analyses shown in Figure 6B. Values are the mean ±SD expressed as fold induction (*, p<0.006 vs. CTR; **, p<0.006

vs. olaparib; *n=3*). C. IB analysis for cl-PARP, cl-caspase 3 and caspase 3 in total extracts of OVCAR-3 cells treated as in B. Tubulin represents the loading control. Right graph represents the blots quantification. Values are the mean ±SD expressed as fold induction (*, $p<0.003$ vs. CTR; **, $p<0.004$ vs. olaparib; *n=3*). D. IB analysis for cl-PARP and γH2A.X in total extracts of MDA-MB-468 breast cancer cells treated as in B. Tubulin represents the loading control. E. IB analysis for cl-PARP, cl-caspase 3, caspase 3 and γH2A.X in total extracts of PMOV10 cells treated with MAC, zibotentan, BQ123 or BQ788 (1µM) for 48h. Tubulin represents the loading control. F. IB analysis for cl-PARP, cl-caspase 3, caspase 3 and γH2A.X in total extracts of PMOV10 cells treated with MAC or zibotentan, alone or in combination with olaparib for 48h. Tubulin represents the loading control. G. Quantification of cl-PARP and γH2A.X in total extracts of PMOV10 cells treated as in B and silenced or not for YAP, HIF-1α or p53 for 72h of IB analyses shown in Figure 6D. Values are the mean ±SD expressed as fold induction (*, $p<0.002$ vs. CTR; **, $p<0.003$ vs. olaparib; *n=3*). H. IB analysis for cl-PARP, and γH2A.X in total extracts of OVCAR-3 cells treated and transfected as in G. Tubulin represents the loading control. Right graph represents the blots quantification. Values are the mean ±SD expressed as fold induction (*, $p<0.009$ vs. CTR; **, $p<0.0004$ vs. olaparib; *n=3*). Representative images of blots of 3 independent experiments are shown in C, D-F and H.
