# Supplementary material for: Targeting tumor-stroma communication by blocking endothelin-1 receptors sensitizes high-grade serous ovarian cancer to PARP inhibition
Source: Cell Death Dis. 2023 Jan 5;14(1):5. doi: 10.1038/s41419-022-05538-6 (PMC9816119; doi:10.1038/s41419-022-05538-6)
Supplement: Supplementary file 2 — Supplementary Information [file 41419_2022_5538_MOESM2_ESM.pdf]

Supplementary Information

Targeting tumor-stroma communication by blocking endothelin-1 receptors sensitizes high-grade serous ovarian cancer to PARP inhibition

Piera Tocci[1*], Celia Roman[1], Rosanna Sestito[1], Valeriana Di Castro[1], Andrea Sacconi[2], Ivan Molineris[3], Francesca Paolini[4], Mariantonia Carosi[5], Giovanni Tonon[6,7], Giovanni Blandino[2] and Anna Bagnato[1*]

[1]Preclinical Models and New Therapeutic Agents Unit, Istituto di Ricovero e Cura a Carattere Scientifico (IRCCS), Regina Elena National Cancer Institute, Rome, Italy; [2]Translational Oncology Research Unit, IRCCS, Regina Elena National Cancer Institute, Rome, Italy; [3]Department of Life Science and System Biology, University of Turin, Turin, Italy; [4]Tumor Immunology and Immunotherapy Unit, IRCCS, Regina Elena National Cancer Institute, Rome, Italy; [5] Pathology Unit, IRCCS, Regina Elena National Cancer Institute, Rome, Italy; [6]Center for Omics Sciences (COSR) and Functional Genomics of Cancer Unit, Division of Experimental Oncology, IRCCS San Raffaele Scientific Institute, Milan, Italy; [7]Università Vita-Salute San Raffaele, 20132, Milan, Italy.

*****Correspondence and requests for materials should be addressed to Anna Bagnato (email: annateresa.bagnato@ifo.it) or to Piera Tocci (email: piera.tocci@ifo.it)

Supplementary Materials and Methods

Silencing experiments

ARRB1, *YAP1* and *LATS1* were knocked-down as previously reported [1], *TP53* and *HIF-1α* were knocked-down using SMART Pool ON-TARGET plus siRNA oligonucleotides: HIF-1α: L-004018-00; p53: L-003329-00 (Dharmacon RNA Technology, CO, USA). In parallel, a pool of 4 non-targeting siRNA was used as negative control (SCR; L-011511-00). Lipofectamine RNAiMAX (Thermo Fisher Scientific, MA, USA) was employed as transfection reagent as instructed by the manufacturer. Silencing efficiency was assessed by IB.

Immunoblotting (IB) and immunoprecipitation (IP)

Protein extracts from whole cell lysates as well as nuclear and cytoplasmic fractions were quantified using a Bio-Rad Protein Assay Kit (Bio-Rad, CA, USA). Anti-histone H3 (1:200, cat. no. sc-8654, Santa Cruz Biotechnology, TX, USA) and anti-tubulin (1:200, DM1A cat. no. sc-32293, Santa Cruz Biotechnology) antibodies (Abs) were utilized as loading controls to assess the purity of the nuclear and cytoplasmic fractions, respectively. Co-IP experiments for endogenous YAP and HIF-1α were carried-out using 200 μg of precleared nuclear cell fractions which were incubated overnight at 4 °C with gentle rotation with anti-YAP (1A12, Cell Signaling Technology, MA, USA), anti-HIF-1α (GT10211, GeneTex, CA, USA) Abs or with anti-rabbit immunoglobulin IgG (ThermoFisher Scientific), or anti-mouse immunoglobulin IgG (Thermo Fisher Scientific) as controls and protein G-Sepharose beads (Cytiva, Sweden). Bound proteins were eluted in SDS loading buffer to subsequent IB. Bands were visualized using the enhanced chemiluminescence (ECL) detection (Bio-Rad). Information regarding Abs used in this research is detailed in Supplementary Table 1 included in the Supplementary Information.

Immunofluorescence (IF) assay

Cells were fixed for 20 min using 4% formaldehyde. Next, specimens were permeabilized in 0.3% Triton for 5 min, blocked in PBS/0.5% BSA for 1 h and incubated overnight at 4°C with primary Abs (anti-phospho-histone H2A.X [γH2A.X] S139, 1:300, Millipore, MA, USA; anti-RAD51, 1:200 Millipore). Then, cells were incubated for 2 h in the dark with the secondary Abs goat anti-mouse Alexa Fluor 488 (1:200, cat. no. A11001, Thermo Fisher Scientific) or goat anti-rabbit Alexa Fluor 594 (1:200, cat. no. A11037, Thermo Fisher Scientific). Nuclei were counterstained with DAPI (Bio-Rad) for 15 min. Slides were visualized at 64X magnification with a Leica DMIRE2 deconvolution microscope equipped with a Leica DFC 350FX camera and elaborated by FW4000 deconvolution software (Leica, Germany).

Proximity ligation assay (PLA)

Cells were fixed for 20 min using 4% formaldehyde. Next, cells were permeabilized in 0.3% Triton for 20 min and blocked in PBS/0.5% BSA for 30 min. After, the cells were incubated overnight at 4 °C with primary abs YAP (G-6) (1:10, cat. no. 376830, Santa Cruz) and HIF-1α (1:10, cat. C166867, LS BIO, WA, USA). The PLA was performed using Duolink In Situ PLA Probe Anti-Mouse PLUS (cat. #DUO92001, Sigma–Aldrich) and Duolink In Situ PLA Probe Anti-Rabbit MINUS (cat. #DUO92005, Sigma–Aldrich) as directed by the manufacturer. Fluorescence signals were captured with a Leica DMIRE2 microscope and quantified with the ImageJ program.

Quantitative real-time PCR (qRT-PCR)

Cells were harvested in TRIzol reagent (Thermo Fisher Scientific) for total RNA extraction. SuperScript® VILO™ cDNA synthesis kit (Thermo Fisher Scientific) was applied to reversely transcribe RNA. *ET-1*, *CTGF* and *VEGF* mRNA expression was profiled using Power SYBR Green PCR Master Mix (Applied Biosystems, CA, USA) with 7500 Fast Real-Time PCR Instrument System (Applied Biosystems). Gene expression values were normalized to cyclophilin-A (*CYPA*) mRNA

expression and 2^ΔΔct method was used to calculate the relative fold gene expression of samples. Primer sequences are reported in Supplementary Table 3 included in the Supplementary Information.

Luciferase assay

Transcriptional activities of ET-1 and VEGF promoters were measured in cells transiently silenced for β-arr1, YAP, p53 or HIF1α for 48 h and co-transfected for 24 h with an ET-1 or a VEGF reporter construct together with a pCMVβ-galactosidase vector (Promega, WI, USA), as previously reported [1]. Luciferase activity was measured using the Luciferase Reporter Assay System (Promega). Each value of luciferase reporter luminescence was normalized to β-galactosidase activity.

Cell vitality analysis

HG-SOC PMOV10 cells were transiently silenced for endogenous YAP, HIF-1α or p53 and treated with macitentan and olaparib, either alone or in combination. After 48 h, cells were stained with trypan blue and then counted with a Neubauer chamber under a microscope.

Chromatin immunoprecipitation (ChIP) assay

Chromatin was analyzed from $5X10^6$ cells by ChIP. The precleared chromatin was incubated overnight on a rotating shaker with anti-β-arr1 (2 μg/μl, E274, cat. no. ab32099, Abcam, United Kingdom), anti-YAP (2 μg/μl, H-125, cat. no. sc-15407, Santa Cruz Biotechnology), anti-p53 (2 μg/μl, DO-1, cat. no. sc-126, Santa Cruz Biotechnology), anti-HIF1α (2 μg/μl, ab243860, Abcam) or anti-mouse IgG Isotype Control (Thermo Fisher Scientific). Co-immunoprecipitated DNA was analyzed by PCR.

Migration and invasion assays

The cell migratory and invasive ability was determined using Transwell migration and matrigel invasion assays. In brief, HUVEC (4 X 10^4), WI-38 (4 X 10^4) and PMOV10 (5 X 10^4) cells were seeded in the upper part of Boyden chambers (BD Biosciences, NJ, USA) and stimulated in the lower part of chambers with serum-free medium alone, in the presence or absence of ET-1, with CM derived from PMOV10 cells, HUVECs or WI-38 cells that were previously silenced for YAP, HIF-1α and p53, or treated with macitentan and olaparib, alone or in combination. Invasion ability was measured by using a Matrigel Invasion Chamber (BD Biosciences). PMOV10 cells (5 X 10^4) transiently silenced for YAP, HIF-1α or p53 were stimulated with serum-free medium, or with ET-1 and/or macitentan or with olaparib, alone or in combination with macitentan added to the lower chamber. After 24 h, the migrating and invading cells were visualized using a Diff-Quick kit (Dade Behring, IL, USA) and detected under a ZOE Fluorescent Cell Imager (Bio-Rad). Migrating and invading cells were counted using the ImageJ program.

Endothelial tube formation assay

HUVEC (3 X 10^4) were seeded in a 96-well culture plate precoated with growth factor reduced matrix (50 µl/well, Cultrex) and stimulated with serum-free medium with ET-1, CM derived from PMOV10 cells previously silenced for YAP, HIF-1α or p53, or treated with macitentan for 4 h. Representative images of tube formations were captured at 20X magnification with a ZOE Fluorescent Cell Imager.

TCGA data analysis

Total RNA was isolated from PMOV10 cells using the RNeasy Quick Start kit (Qiagen, Germany) according to manufacturer's instructions. NanoDrop (Thermo Scientific) was used to check RNA concentration, quality and purity. Before starting with library prep, RNA quality was determined by running samples on the 4100 TapeStation system (Agilent, CA, USA). Only RNA samples with RIN above 7 were used in the next steps. Libraries were barcoded, pooled, and sequenced on an Illumina

NextSeq 500 sequencing system to obtain ~ 20-30 million (M) single-end reads, 100 nt long, for each sample. Sequencing reads were aligned to the human reference genome GRCm38 using STAR v2.5.3a [2] (with parameters --outFilterMismatchNmax 999 --outFilterMismatchNoverLmax 0.04) and a list of known splice sites extracted from GENCODE 24 comprehensive annotation. Gene expression levels were quantified with featureCounts v1.6.3 [3] (options: -t exon -g gene_name) using GENCODE 24 basic gene annotation. Next, gene expression counts were analyzed using the edgeR package [4]. Normalization factors were performed using the trimmed-mean of M-values (TMM) method (implemented in the calcNormFactors function), and normalized library sizes and gene lengths were used to obtain RPKM. Upon filtering low-ranking expressed genes (below 1 CPM in 4 or more samples), differential expression analysis was conducted by fitting a GLM to all groups, performing an LF test for the interesting pairwise contrasts. Genes were considered significantly differentially expressed when |logFC| >1 and FDR < 0.05 in each relevant comparison. To generate the NGS libraries, we used the TruSeq Stranded mRNA kit (Illumina, CA, USA), starting with 100 ng of total RNA. GSEA preranked analysis of signaling pathways from Hallmark and Kyoto Encyclopedia of Genes and Genomes (KEGG) gene sets was performed using the Java version of the software (gsea2–2.2.3.jar; software.broadinstitute.org/gsea/), with log2FC obtained from RNA-Seq contrasts and standard parameters.

NOTE:

https://bitbucket.org/cosrhsr/rnaseq/raw/7b95f116bf4b669d4e879b80d6a7f77f787ded8d/local/share/data/metadata_180401_both_runs.txt

https://bitbucket.org/cosrhsr/rnaseq/src/BagnatoA_595_RNASeq_Stranded/local/share/makefiles/makefile_nuova_run2

Cytokine and chemokine assay

PMOV10 cells were seeded (15 X 10^4) and subsequently silenced or not for YAP, HIF-1α or p53 for 72 h and either stimulated or not stimulated with ET-1 for 24 h. Supernatants were collected after 24

h and centrifuged for 10 min at 1000 g to eliminate cell debris and thereafter diluted for the Luminex assay. Briefly, capture antibody-coupled beads were allowed to react with duplicated samples of the diluted supernatants. After performing a series of washes to remove unbound materials, a biotinylated detection antibody specific for a different epitope on the target was added to the beads. The reaction mixture was detected by streptavidin-phycoerythrin, which binds sandwich complexes via biotinylated detection antibody. Cytokines and chemokines were analyzed using a Bio-Plex Pro Human Cytokine Screening 48-Plex panel (Bio-Rad) according to the manufacturer's instructions. The cytokine/chemokine contents of each well were identified and quantified against standard samples using a Bio-Plex Magpix apparatus (Bio-Rad).

Supplementary references

1. Tocci P, Cianfrocca R, Di Castro V, Rosanò L, Sacconi A, Donzelli S, et al. β-arrestin1/YAP/mutant p53 complexes orchestrate the endothelin A receptor signaling in high-grade serous ovarian cancer. Nat Commun. 2019;10:3196.

2. Dobin A, Davis CA, Schlesinger F, Drenkow J, Zaleski C, Jha S, et al. STAR: ultrafastuniversal RNA-seqaligner. Bioinformatics. 2013;29:15-21.

3. Liao Y, Smyth GK, Shi W. Feature Counts: an efficient general purpose program for assigning sequence reads to genomic features. Bioinformatics. 2014;9:23-30.

4. Robinson MD, McCarthy DJ, Smyth GK. edgeR: a Bioconductor package for differential expression analysis of digital gene expression data. Bioinformatics. 2013;26:139-140.

Supplementary Tables

**Supplementary Table 1. Antibodies used for IB, IP and ChIP experiments**		
**Antigen**	**Dilution**	**Manufacturer**
Tubulin (DM1A) cat. no. sc-32293	1:200	Santa Cruz Biotechnology
pYAP (S127) D9W2I cat. no. 13008	1:1000	Cell Signaling
YAP 1A12 cat. no. 12395	1:1000	Cell Signaling
YAP (G6) cat. no. sc-376830	1:1000	Santa Cruz
YAP (H-125) cat. no. sc-15407	1:1000	Santa Cruz
β-arrestin 1 E274 cat. no. ab32099	1:500	Abcam
p53 (DO1) cat. no. sc-126	1:200	Santa Cruz Biotechnology
Cleaved-PARP (Asp 214) cat. no. 9541	1:1000	Cell Signaling
RAD 51 cat. no. ABE257	1:200	Millipore
pγH2A.X (S139) cat. no. 05-636	1:200	Millipore
Caspase 3 (D3R6Y) cat. no. 142200	1:1000	Cell Signalling
β-actin (AC-15) cat. no. #1978	1:5000	Sigma-Aldrich
HIF-1α cat. no. 610959	1:1000	BD Biosciences
HIF-1α (GT10211) cat. no. GTX628480	1:1000	GeneTex
HIF-1α cat. no. ab243860	1:500	Abcam
Histone H3 (D1H2) XP cat. no. 4499	1:2000	Cell Signalling
VEGF cat. no. MAB293	1:1000	R&D Systems
VEGF (VG-1) cat. no. sc-53462	1:200	Santa Cruz Biotechnology
PCNA (F-2) cat. no. sc-25280	1:200	Santa Cruz Biotechnology
α-SMA (D4K9N) XP cat. no. 19245S	1:1000	Cell Signalling
Vimentin (D21H3) XP (R) cat. no. 5741S	1:1000	Cell Signalling
Fibronectin cat. no. F2518	1:25	SIGMA-ALDRICH
pLATS1 (T1079) D57D3 cat. no. 8654	1:1000	Cell Signaling
LATS1 cat. no. A300-477A	1:5000	Bethyl

Supplementary Table 2. Primers used for ChIP experiments	
**Promoter**	**Primer sequences**
ET-1 F	5′-CAGCTTGCAAAGGGGAAGCG-3′
ET-1 R	5′- TCCGACTTTATTCCAGCCCC -3’
VEGF F	5’-AGGAACAAGGGCCTCTGTCT-3’
VEGF R	5’-CAGTGTGTCCCTCTGACAATG-3’

Supplementary Table 3. Primers used for qRT-PCR experiments	
**Target gene**	**Primer sequences**
CTGF F	5’-AGGAGTGGGTGTGTGACGA-3’
CTGF R	5’-CCAGGCAGTTGGCTCTAATC-3’
EDN1 F	5’-CCAAGAGAGCCTTGGAGAAT-3’
EDN1 R	5’-TGTCTTCAGCCCTGAGTTCTT-3’
VEGF F	5’-ACAGACGTTCCTTAGTGCTGG-3’
VEGF R	5’-AGCTGAGAACGGGAAGCTGTG-3’
CYCLOPHILIN-A F	5’-TTCATCTGCACTGCCAAGAC-3’
CYCLOPHILIN-A R	5’-TCGAGTTGTCCACAGTCAGC-3’
