# Supplementary material for: Targeting tumor-stroma communication by blocking endothelin-1 receptors sensitizes high-grade serous ovarian cancer to PARP inhibition
Source: Cell Death Dis. 2023 Jan 5;14(1):5. doi: 10.1038/s41419-022-05538-6 (PMC9816119; doi:10.1038/s41419-022-05538-6)

Uncropped Blots Fig. 1

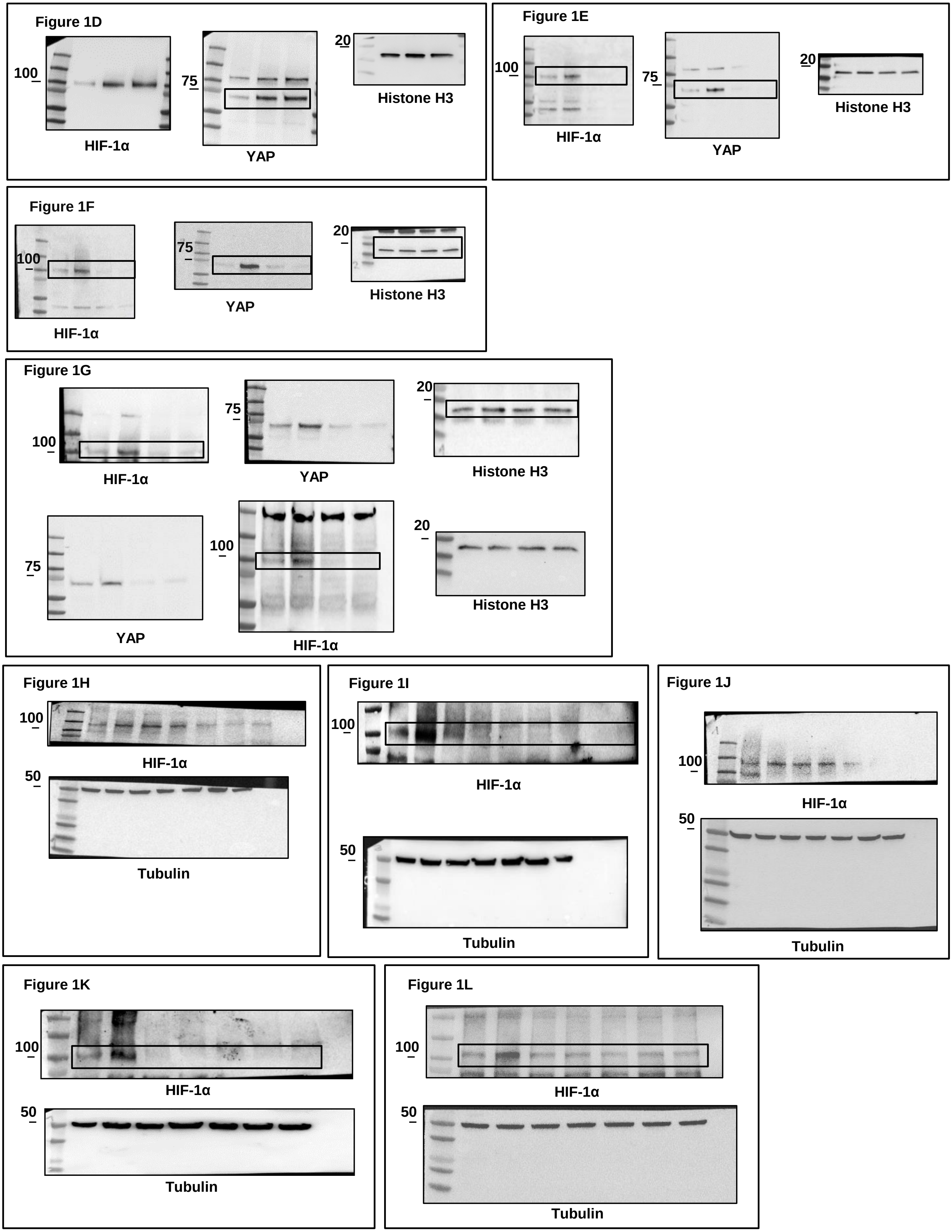

Uncropped Blots Fig. 2

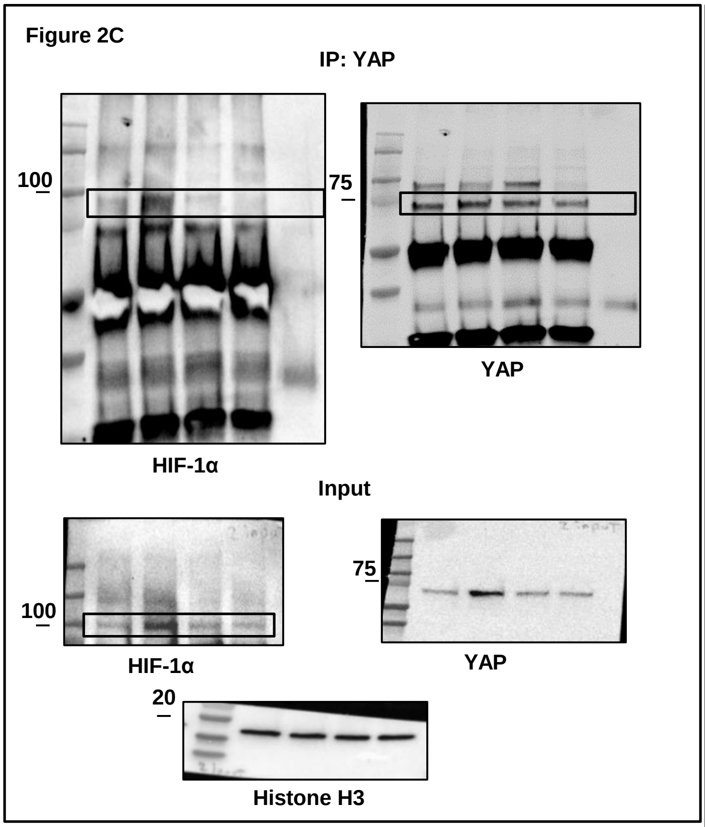

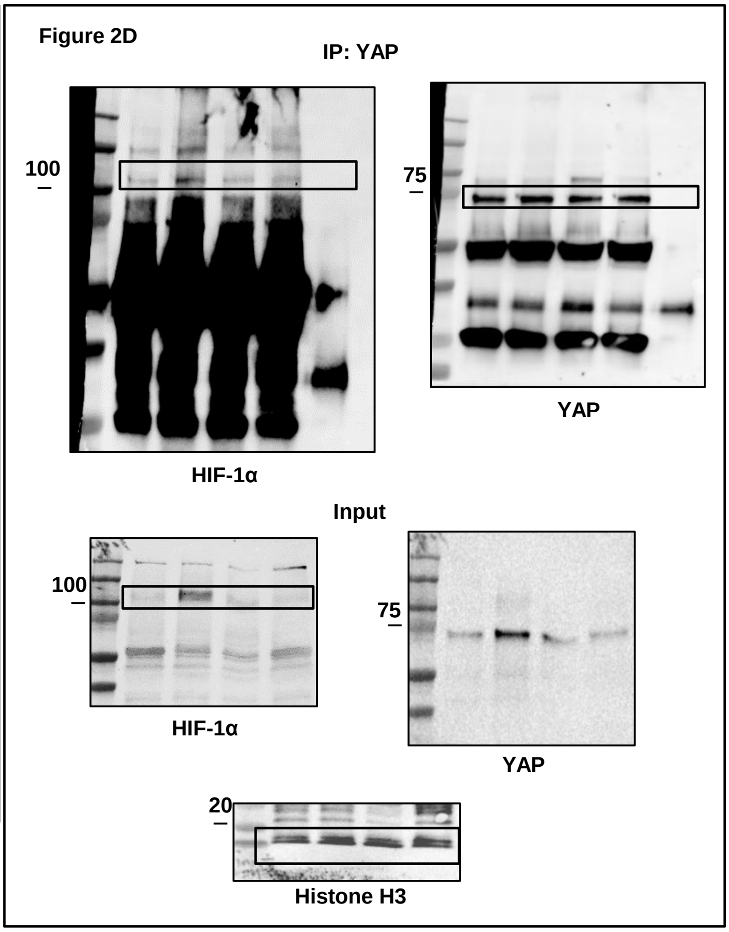

Uncropped Blots Fig. 3-4

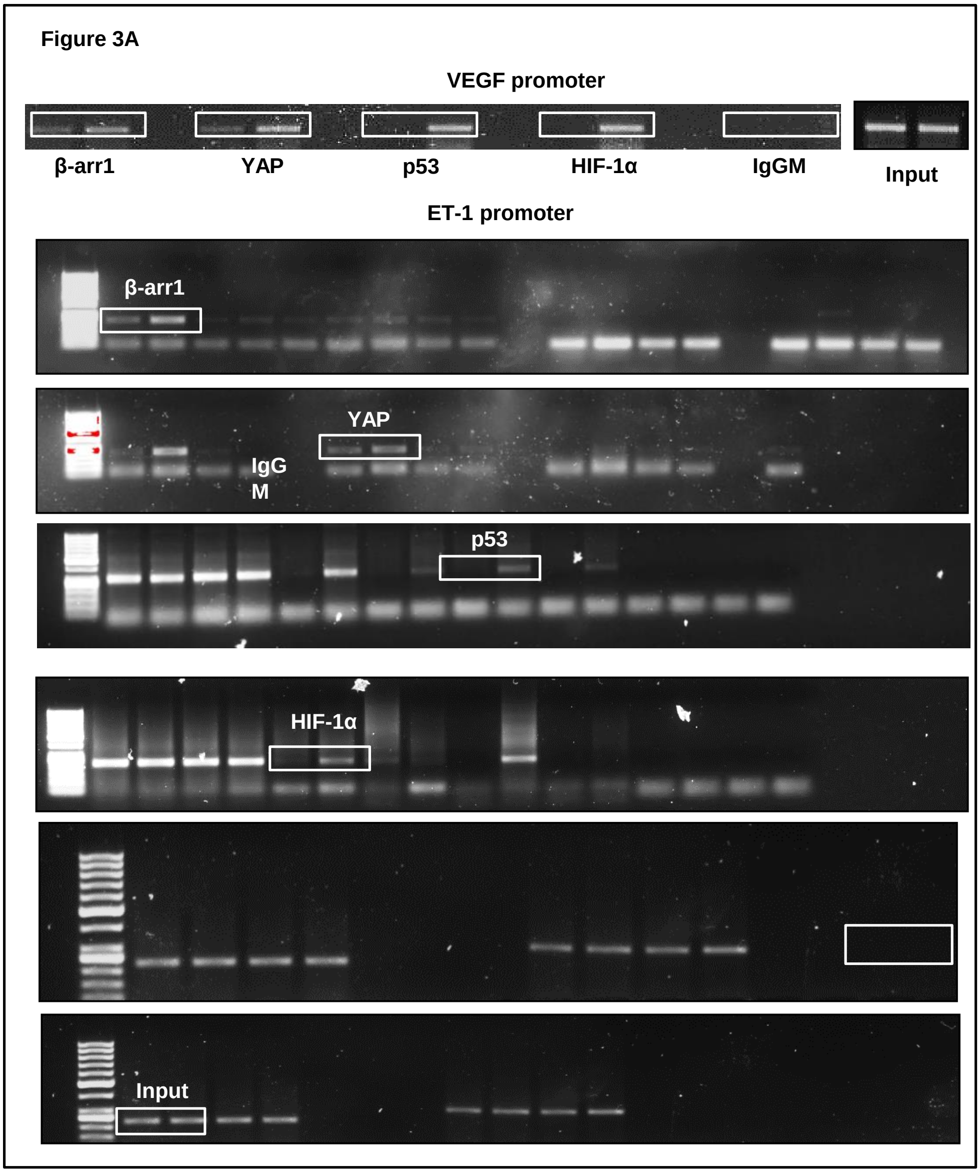

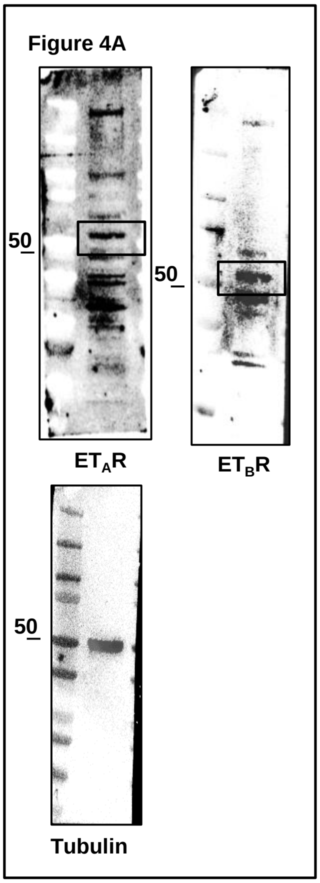

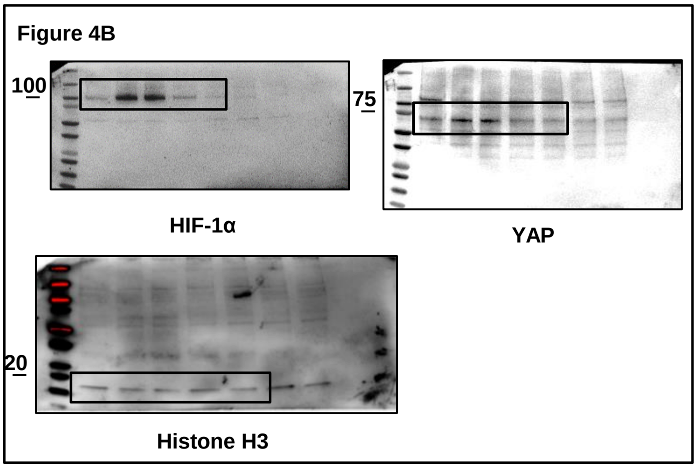

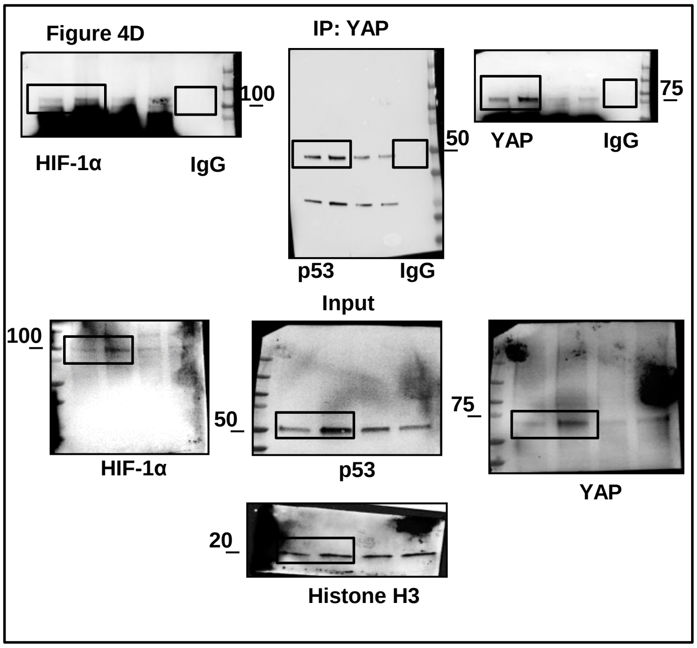

Uncropped Blots Fig. 5

Figure 5A

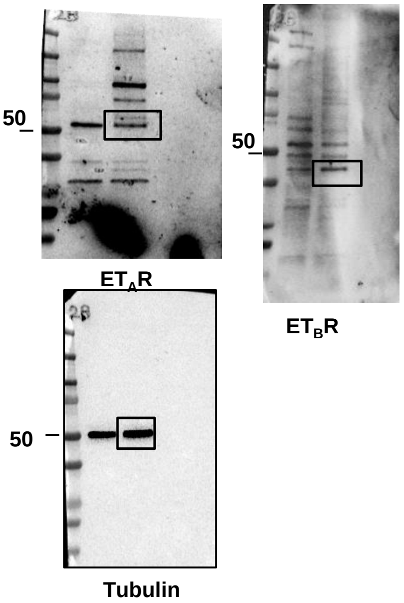

Figure 5B

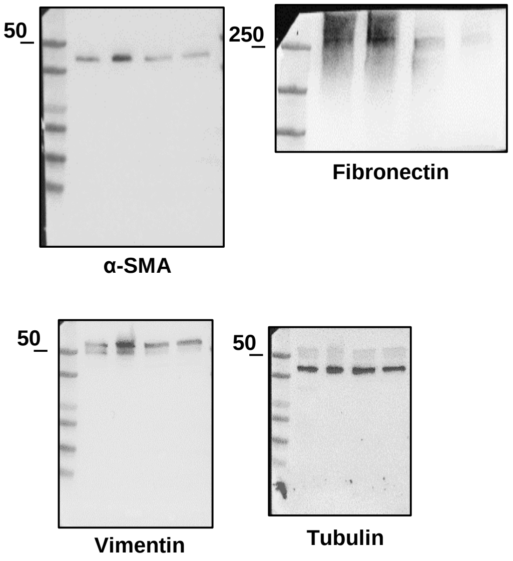

Figure 5C

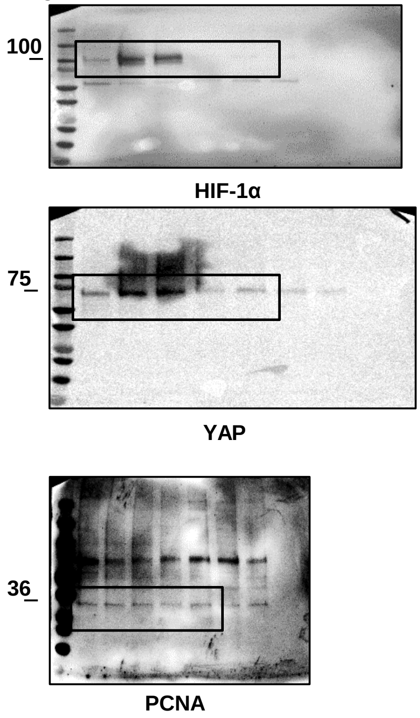

Figure 5E

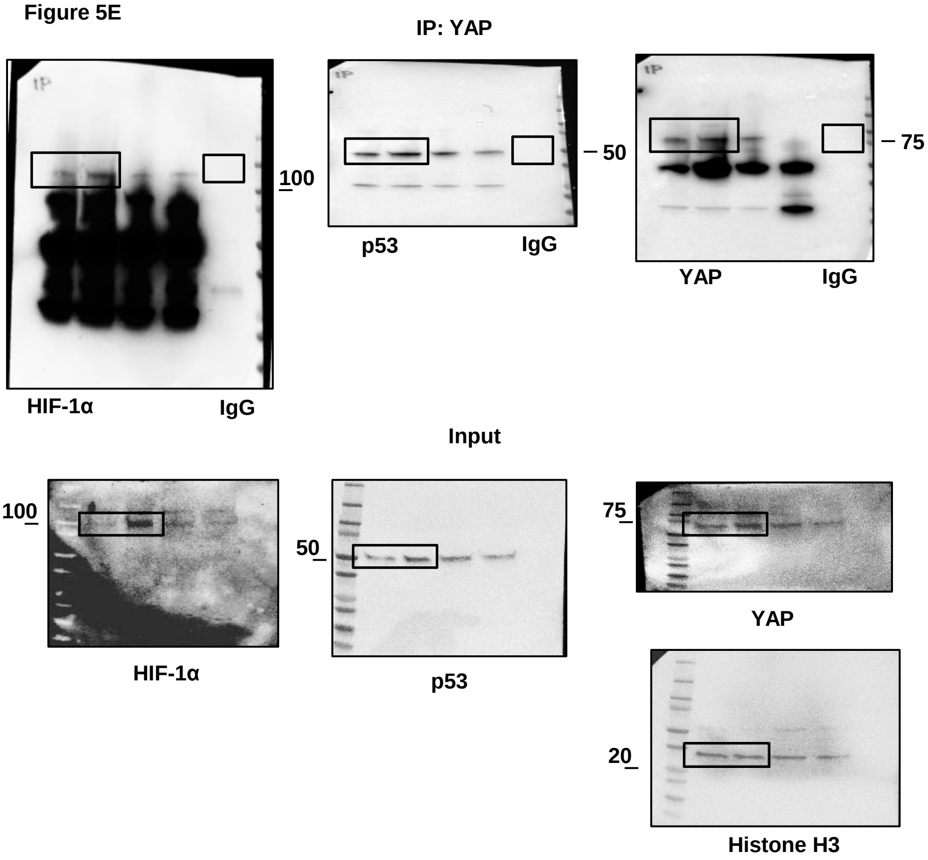

Uncropped Blots Fig. 6-7

Figure 6B

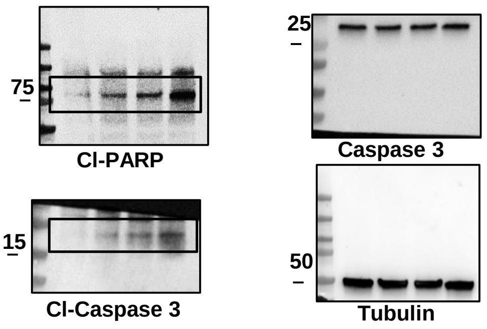

Figure 6D

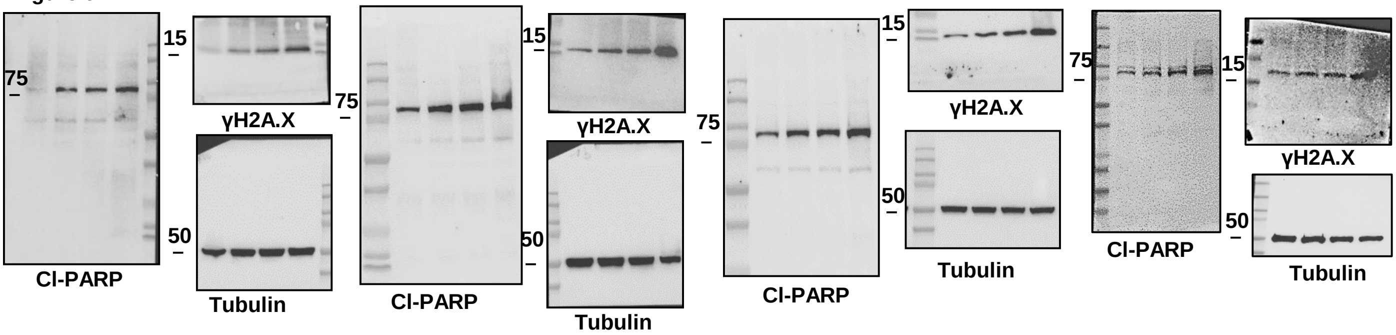

Figure 7B

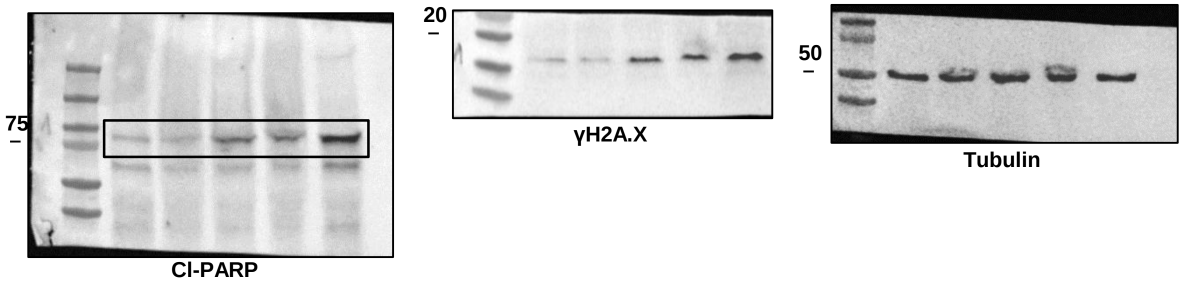

Figure 7C

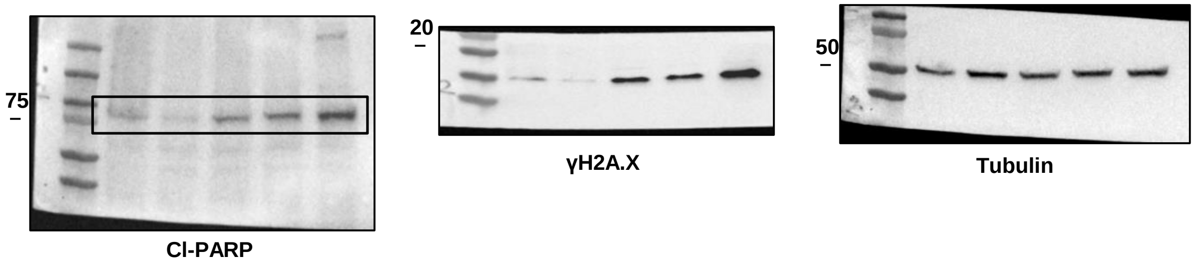

Uncropped Blots Fig. 6-8

Figure 8C

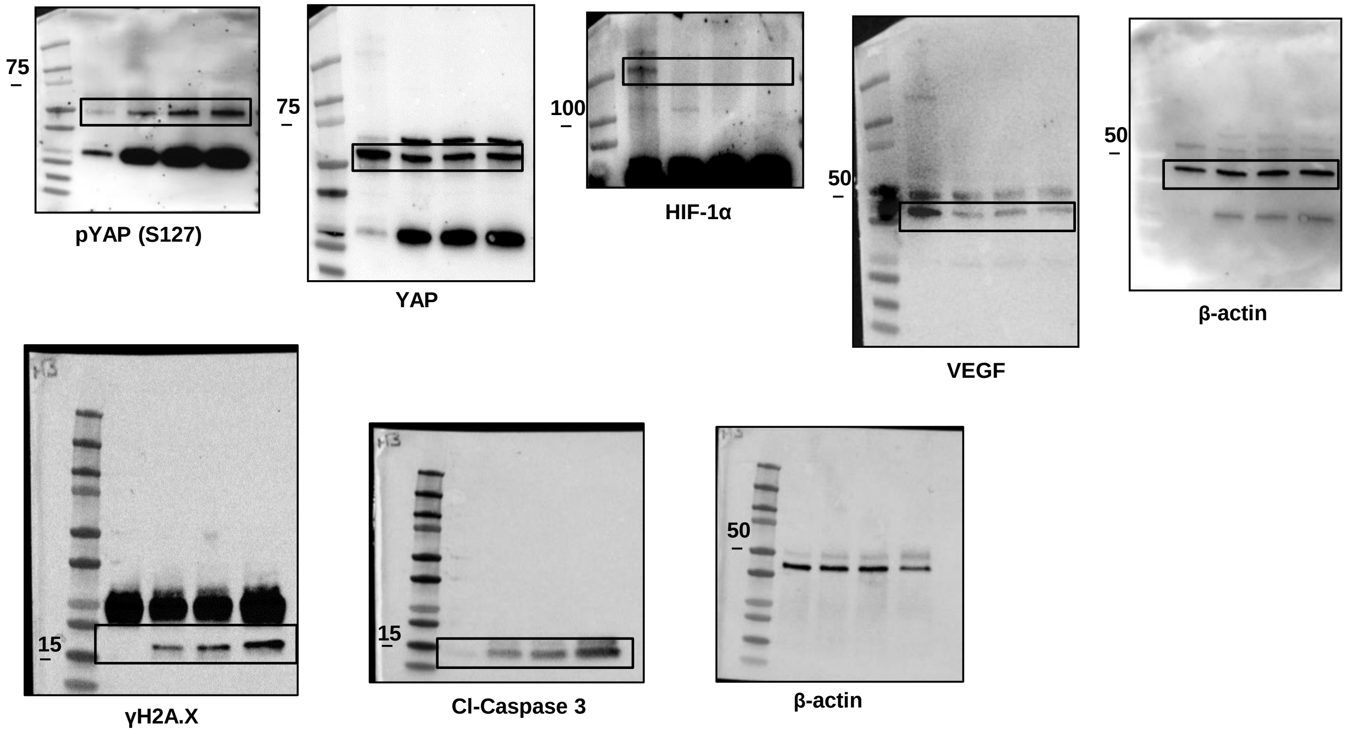

Figure 8E

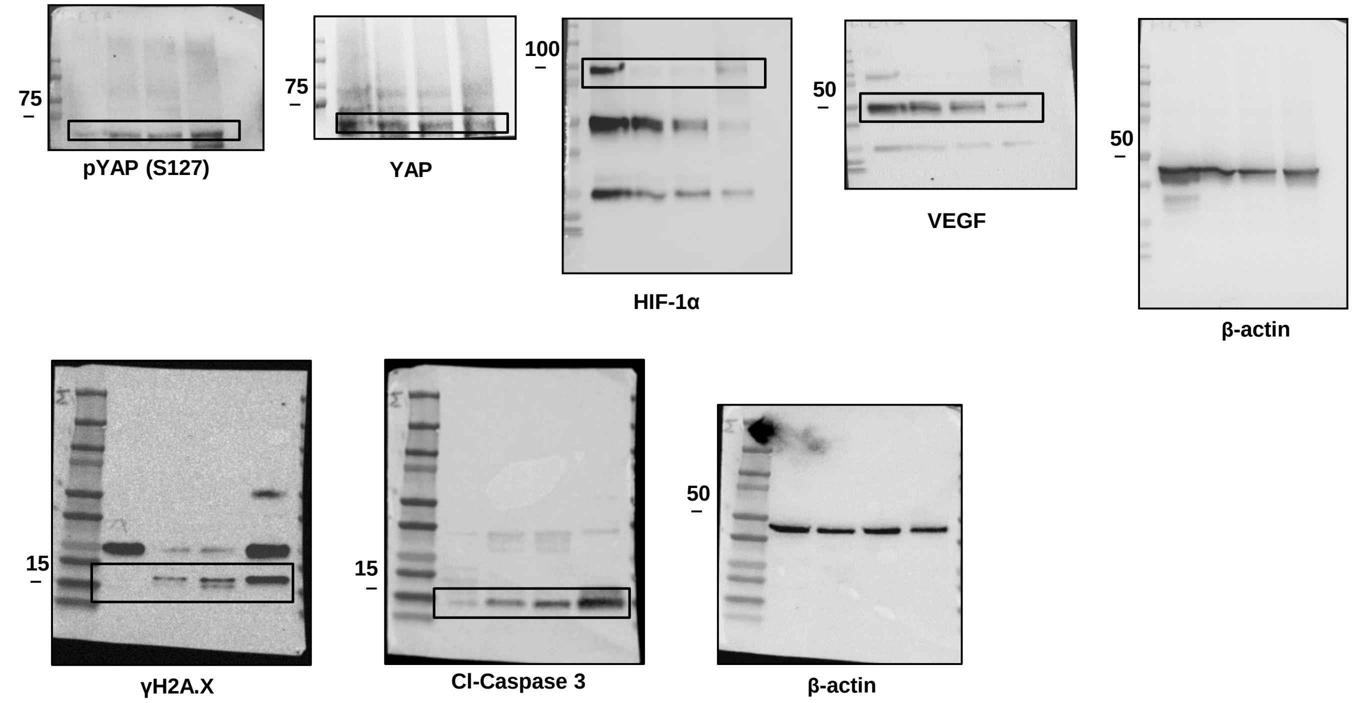

Uncropped Blots Supplementary Fig. 1

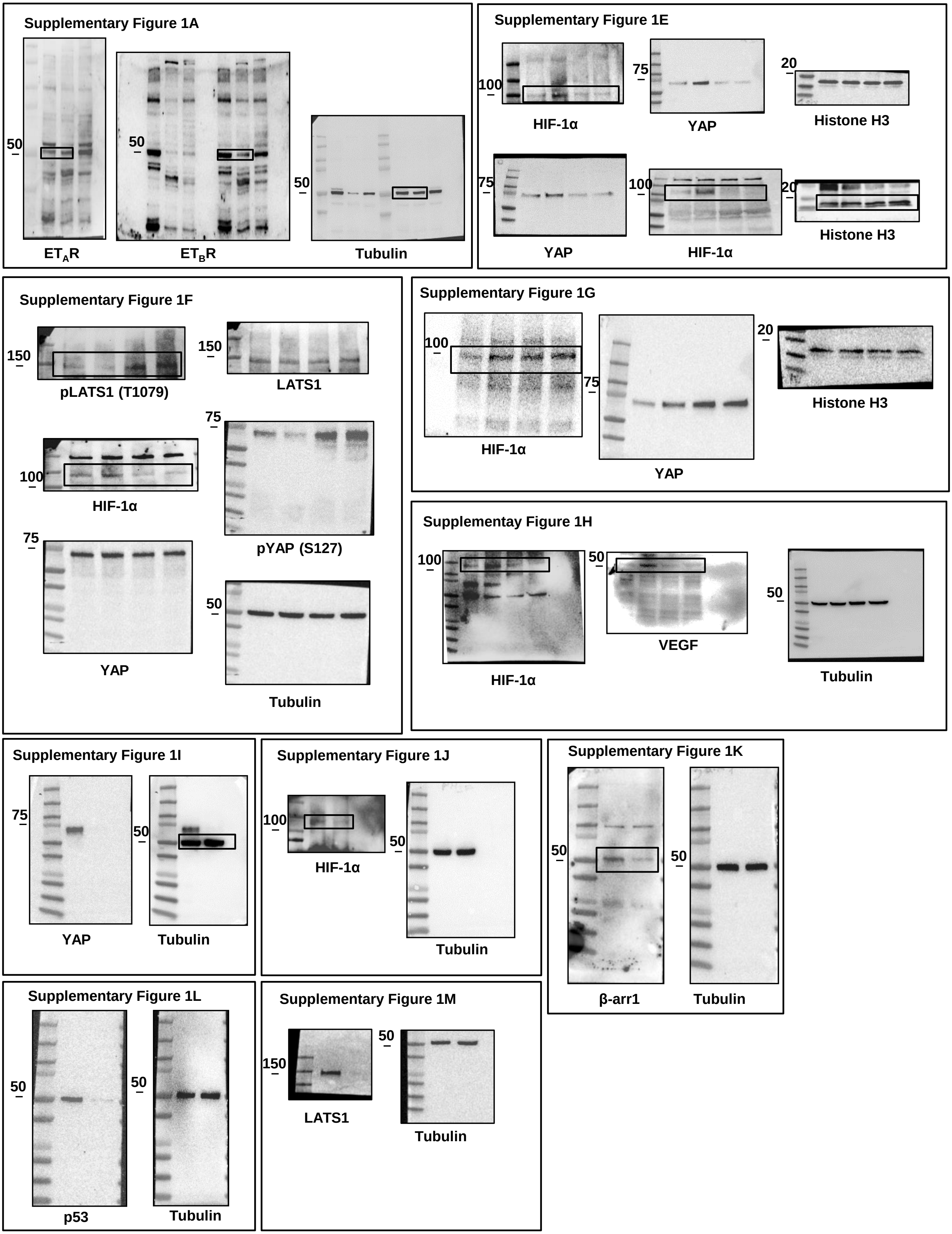

Uncropped Blots Supplementary Fig. 2

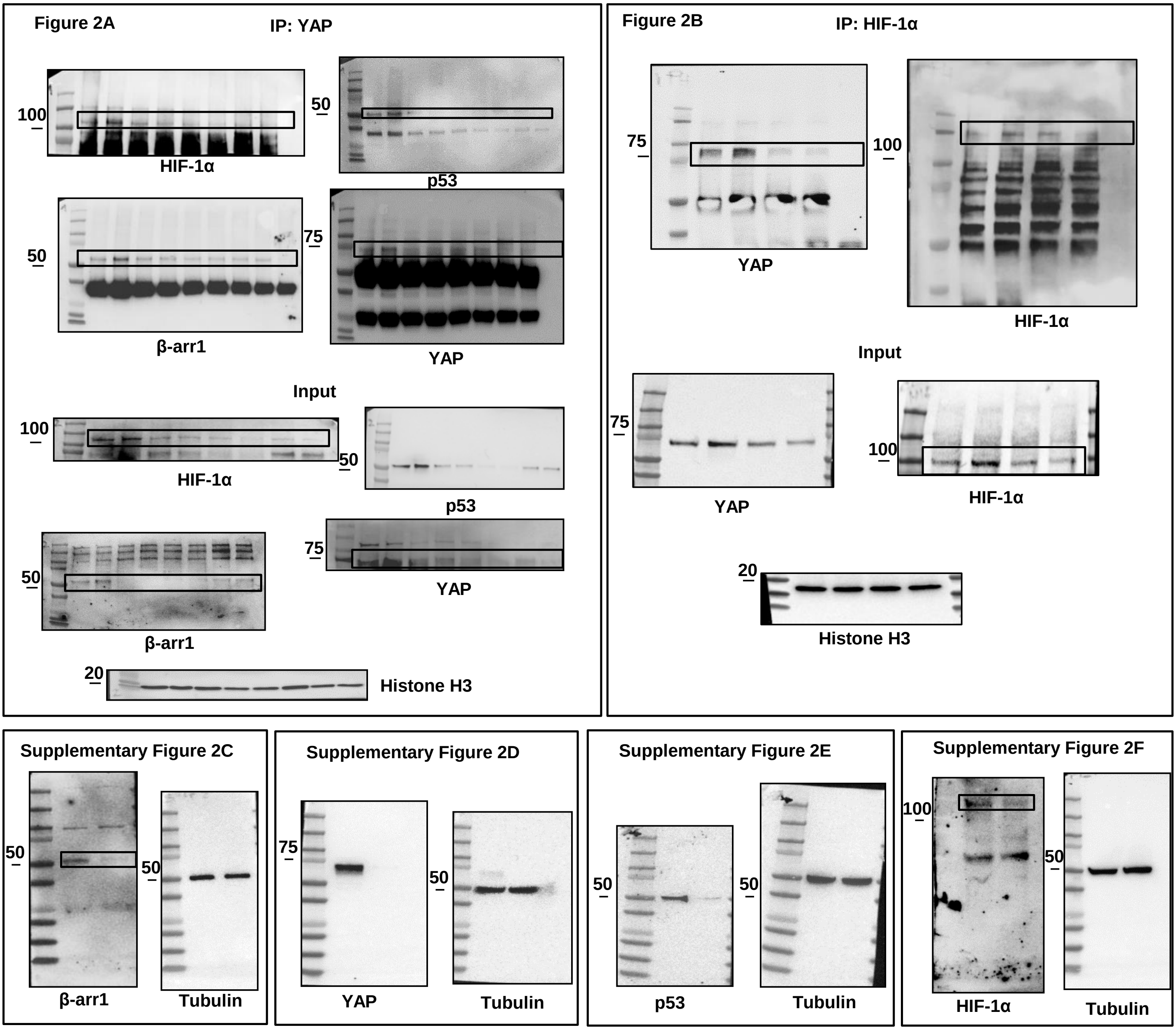

Uncropped Blots Supplementary Fig. 4-5

Supplementary Figure 4A

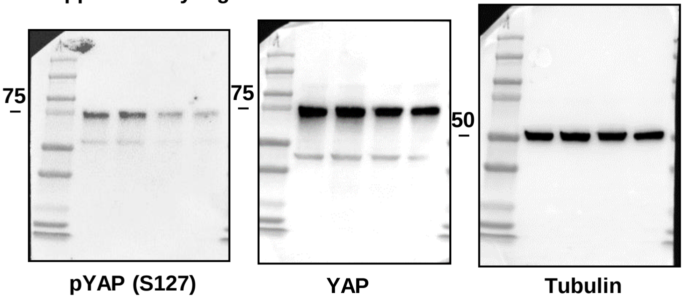

Supplementary Figure 4B

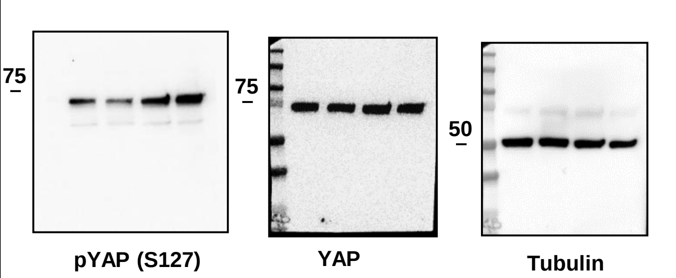

Supplementary Figure 4C

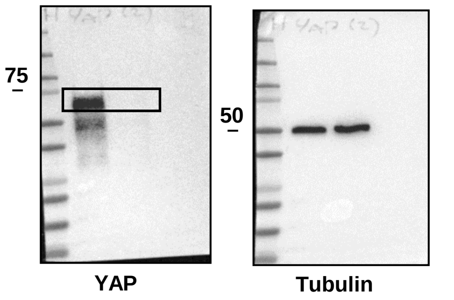

Supplementary Figure 4D

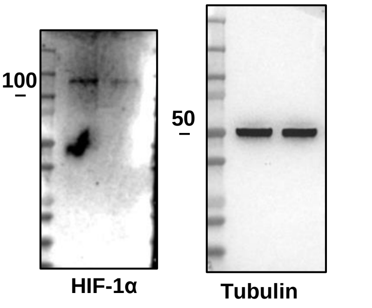

Supplementary Figure 4E

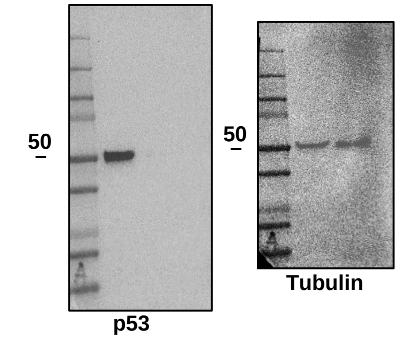

Supplementary Figure 5A

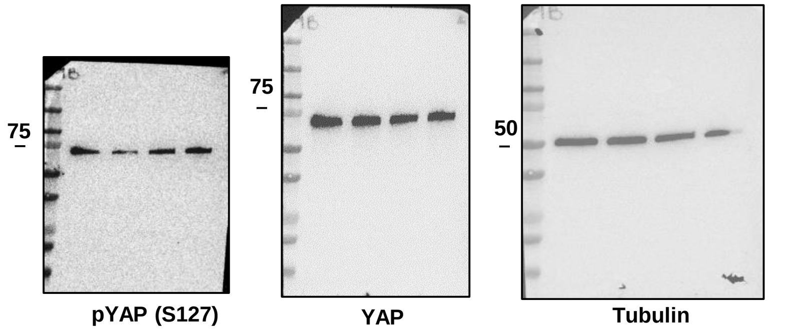

Supplementary Figure 5B

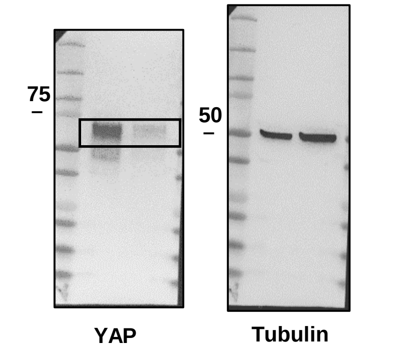

Supplementary Figure 5C

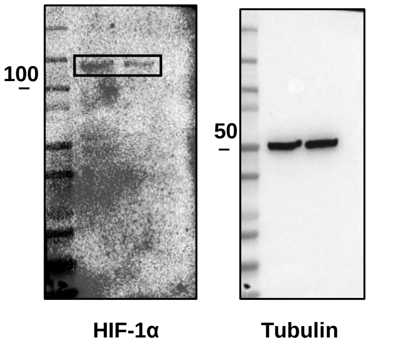

Supplementary Figure 5D

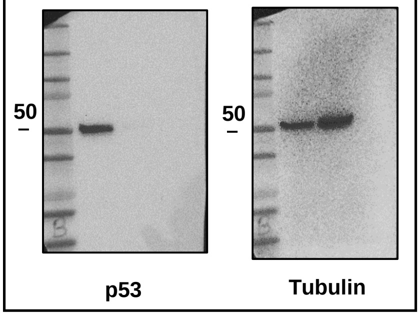

Uncropped Blots Supplementary Fig. 6

Supplementary Figure 6C

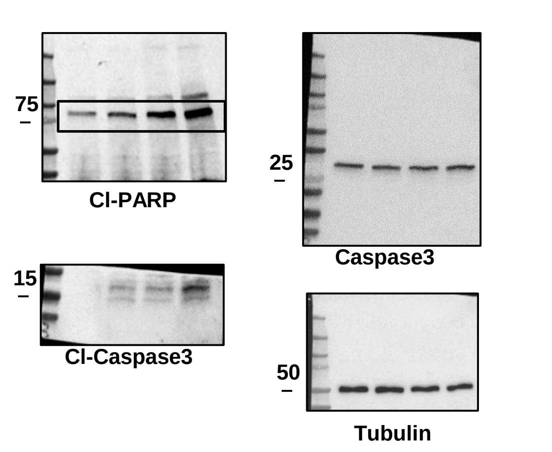

Supplementary Figure 6D

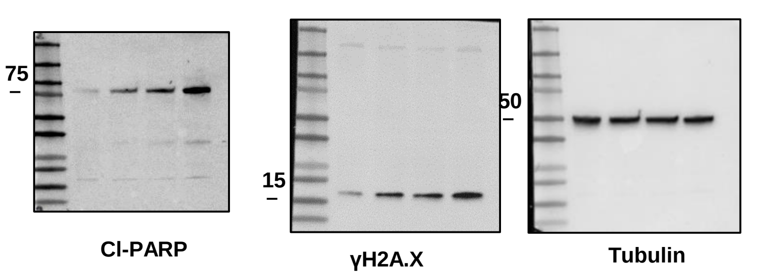

Supplementary Figure 6E

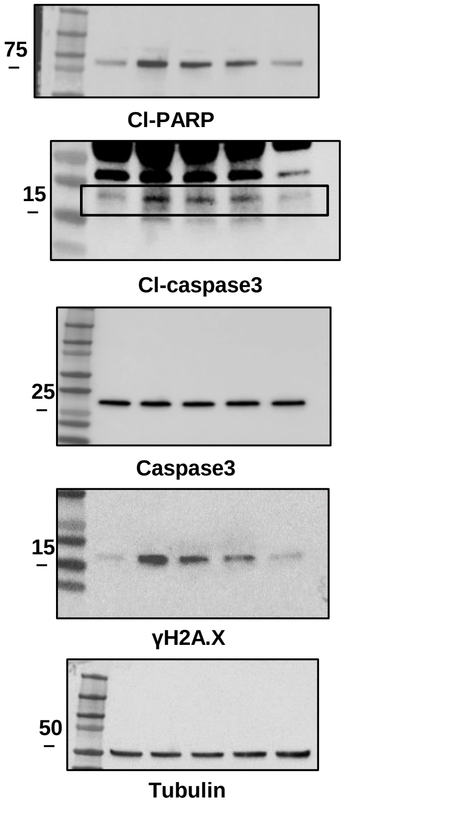

Supplementary Figure 6H

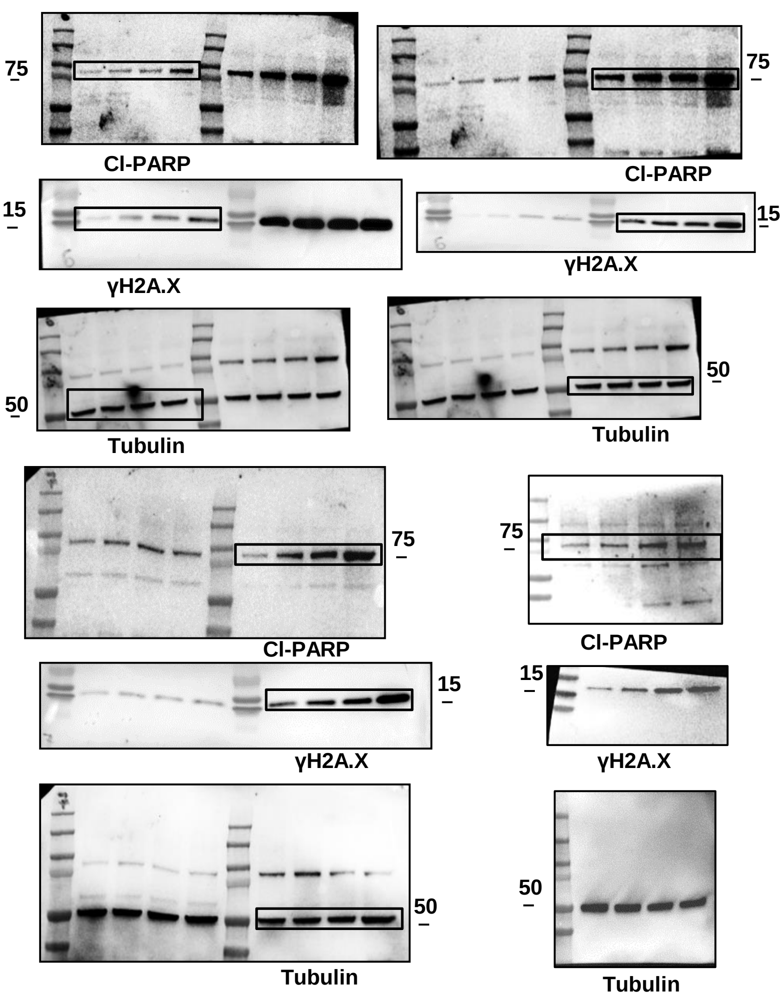

Supplementary Figure 6F

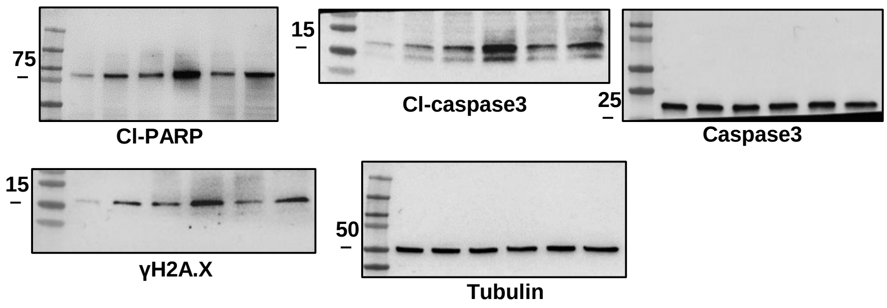

Supplement: Supplementary file 3 — Original Data File [file 41419_2022_5538_MOESM3_ESM.pdf]
